# Supplementary material for: Trainable subnetworks reveal insights into structure knowledge organization in protein language models
Source: PLoS Comput Biol. 2026 Feb 9;22(2):e1013925. doi: 10.1371/journal.pcbi.1013925 (PMC12928587; doi:10.1371/journal.pcbi.1013925)
Supplement: S1 Appendix — Description of learning hyperparameters and compute. (PDF) [file pcbi.1013925.s001.pdf]

Subnetwork training details

For each PLM, we conducted a grid search to select optimal hyperparameter values to achieve suppression in all subnetworks. We set the below hyperparameters to the same values across all subnetworks learned within the same model. We include the results of the grid search on subnetwork performance in the accompanying code and data (see https://github.com/microsoft/plm_subnetworks for more information). The hyperparameters that vary by PLM are listed below and summarized in S1 Table.

- **Mask initialization (s_{init}).** Each mask logit l_i is initialized such that $\sigma(l_i) = p_{\text{keep},0}$, where $p_{\text{keep},0}$ is the desired initial retention probability (e.g., 0.96) of the i-th parameter. This is achieved by setting $l_i = \log\left[p_{\text{keep},0}/(1 - p_{\text{keep},0})\right]$, so that each parameter begins training with a high probability of being retained. This initialization enables the model to start from a near-complete network and learn to suppress parameters selectively over time. We choose $p_{\text{keep},0}$ to ensure that the subnetwork MLM loss initially matches the PLM average loss over all inputs, while introducing less than approximately 1% sparsity. This yields a mask score distribution centered near zero, placing most sigmoid activations in a high-sensitivity regime and allowing flexible updates toward maintenance and suppression goals. We explored the effects of this random initialization of mask scores in S2 Fig.
- **Suppression-KL loss weight ($\lambda_{\text{supp.}}$).** Applied using Eq. (1) and Eq. (2).
- **Maintenance-KL loss weight ($\lambda_{\text{maint.}}$).** Applied using Eq. (3) and Eq. (4).
- **Maintenance-MLM loss weight (λ_{MLM}).** Applied using Eq. (5) and Eq. (6).
- **Mask temperature (τ).** We apply a cosine decay schedule to the temperature τ, decreasing it smoothly from a maximum of τ_{init} to τ_{final} over 100 epochs. This schedule enables high-entropy exploration early in training and progressively sharper binarization of the mask as training progresses.
- **Mask threshold (T).** In practice, the binarization threshold T in Eq. (9) varies by PLM.

Learning rate

We use a maximum learning rate of `peak_lr` with a reverse OneCycle learning rate schedule. The learning rate remains constant at `peak_lr` during an initial plateau phase, defined by `1 / lr_plateau_epochs`, and then decays to `peak_lr` divided by 30 using cosine annealing. The learning rate schedule spans the full number of estimated training steps, as determined by PyTorch Lightning. Only mask parameters are optimized, using the AdamW optimizer with $\beta = (0.9, 0.999)$, $\epsilon = 10^{-6}$, and zero weight decay. The maximum value of `peak_lr` observed in training is 10^{-1}.

Compute

We train each subnetwork on one NVIDIA H100 GPU. We curate training batches of size 16 with a 0.25 frequency of suppression inputs in a batch such that subnetworks training converges over fewer epochs. We report the number of suppression inputs per category in S3 Table and the number training steps in S4 Table.
