# Supplementary figures and images for: Trainable subnetworks reveal insights into structure knowledge organization in protein language models
Source: PLoS Comput Biol. 2026 Feb 9;22(2):e1013925. doi: 10.1371/journal.pcbi.1013925 (PMC12928587; doi:10.1371/journal.pcbi.1013925)

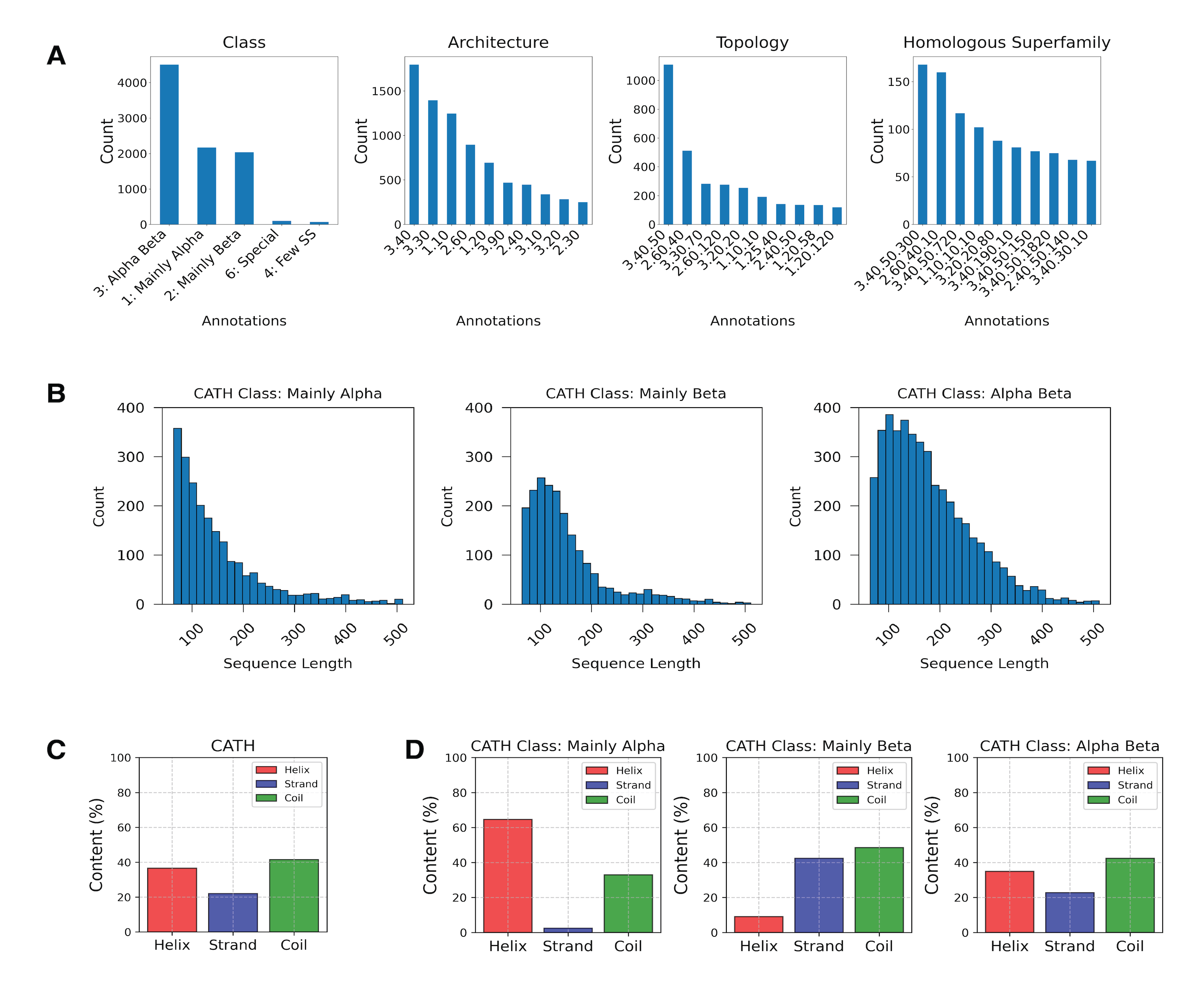

Supplement: S1 Fig — Three subnetworks were independently trained for each CATH Class suppression target (Mainly Alpha, Mainly Beta, Alpha-Beta) to assess the reproducibility of mask learning given random initialization of mask scores. Each point represents the validation perplexity of a subnetwork stratified by category of inputs. (A) Annotation frequencies by CATH levels. Each CATH domain is annotated with a label at the Class, Architecture, Topology, and Homologous Superfamily levels. Bar plots show the counts (y-axis) of the top 10 most frequent annotations (x-axis) at each level of the CATH hierarchy. (B) Sequence length distributions stratified by CATH level. Histograms show the counts (y-axis) of domain sequence lengths (x-axis) for each CATH Class: Mainly Alpha, Mainly Beta, Alpha Beta. (C) Secondary structure composition of all CATH domains. Average fraction of residues annotated as helix, strand, or coil across all CATH domains, based on DSSP annotations [28]. (D) Secondary structure composition stratified by CATH class. DSSP 8-state annotations are mapped to 3-state labels: H, G, I → H; E, B → E; T, S, - → L. Helix is H, beta strand is E, and loop is L. (TIFF) [file pcbi.1013925.s002.tiff]

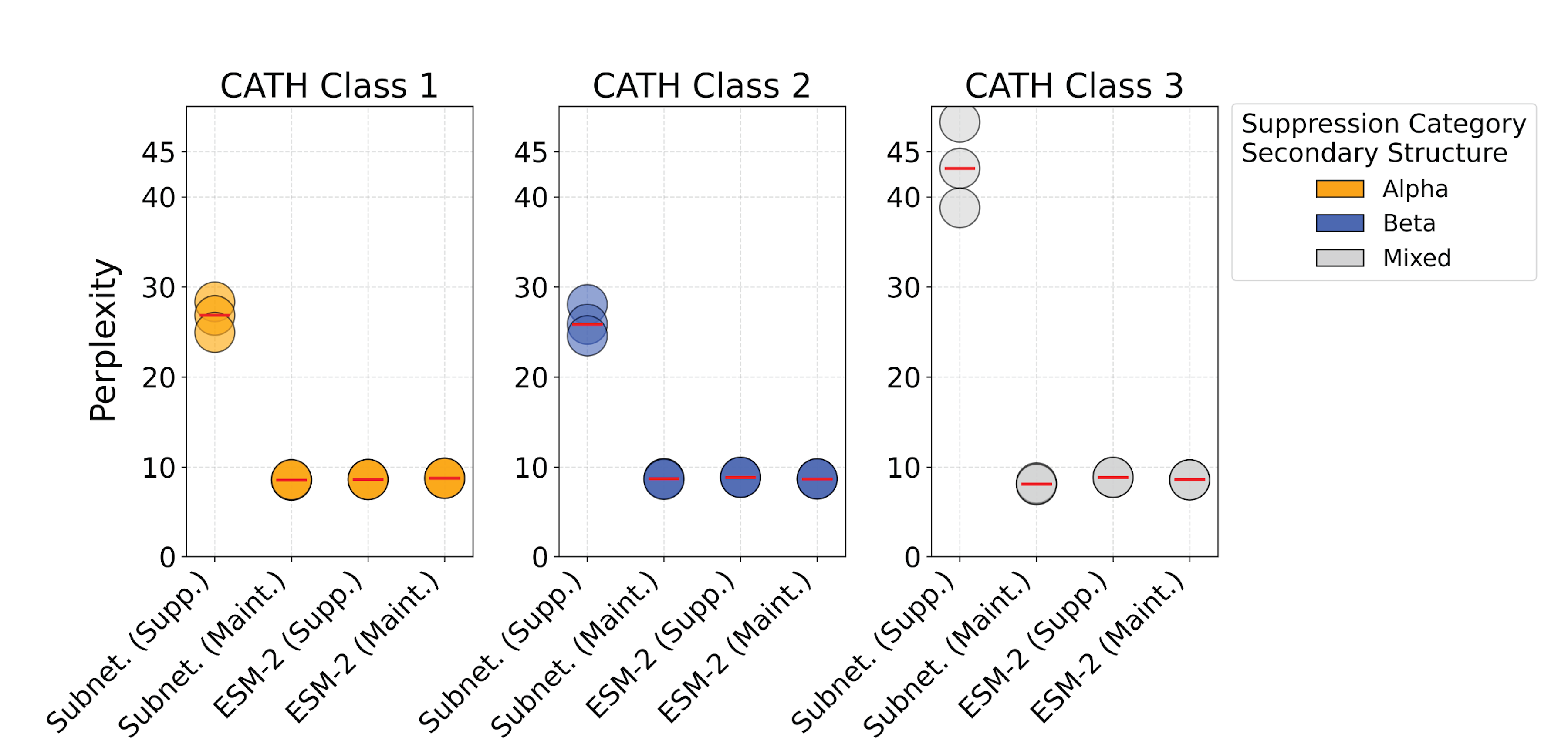

Supplement: S2 Fig — Three subnetworks were independently trained for each CATH Class suppression target (Mainly Alpha, Mainly Beta, Alpha-Beta) to assess the reproducibility of mask learning given random initialization of mask scores. Each point represents the validation perplexity of a subnetwork stratified by category of inputs. (TIFF) [file pcbi.1013925.s003.tiff]

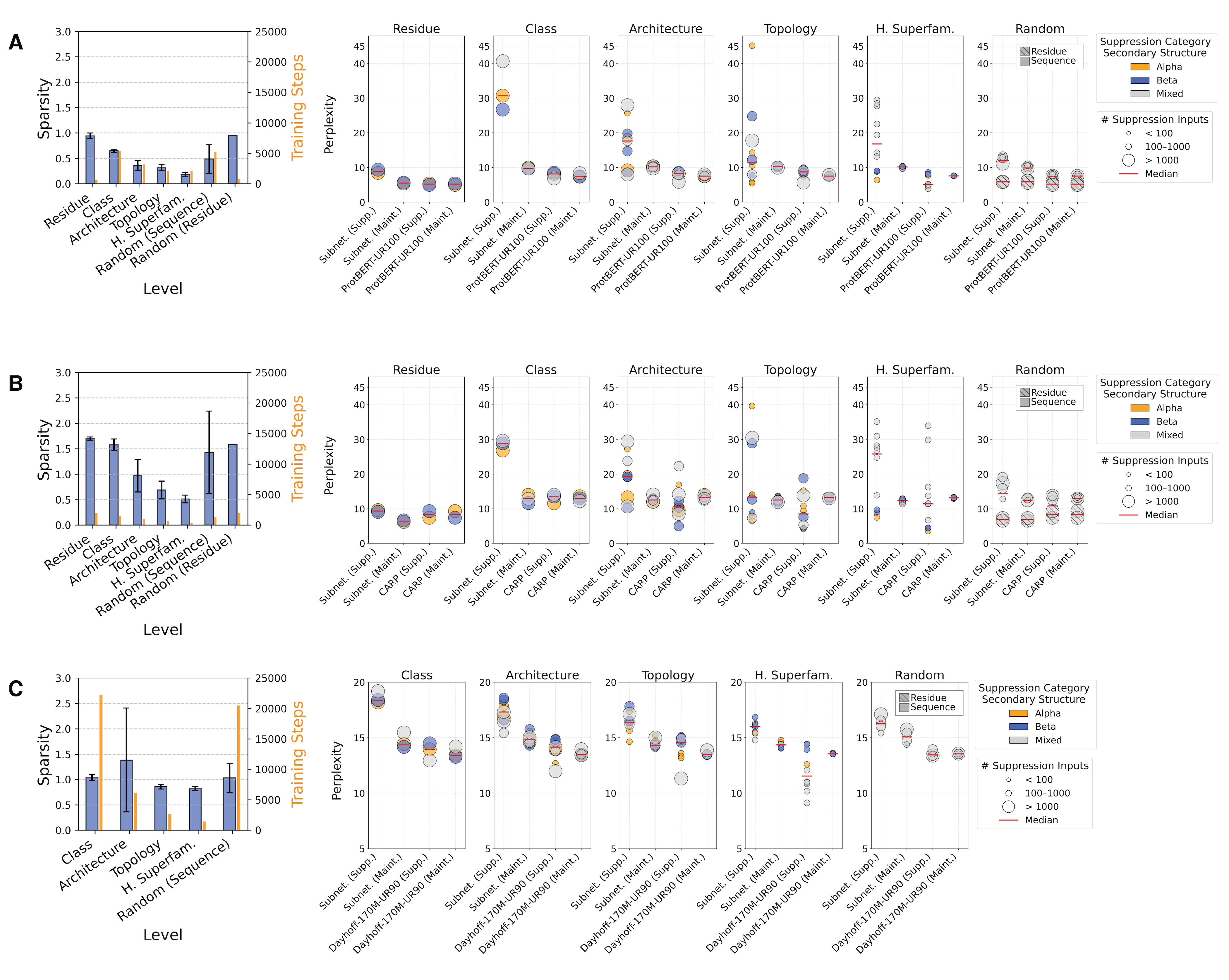

Supplement: S3 Fig — To investigate whether subnetworks exist in pretrained PLMs other than ESM-2, we applied our approach to three additional models of varying size and architectures trained on UniRef data: ProtBERT-UR100, a transformer masked language model with a BERT architecture [2]; CARP-640M, a convolutional neural network masked language model (transformer layers are replaced by ByteNet dilated CNN blocks) [14]; and Dayhoff-170M-UR90, an efficient hybrid state-space-model transformer trained with and autoregressive objective [31]. (A) ProtBERT-UR100 (420M). Left: Learned percent sparsity by category of learned subnetworks in ProtBERT-UR100. Right: Masked language modeling performance on suppression and maintenance categories of sequences for each subnetwork. (B) CARP-640M. Left: Learned percent sparsity by category of learned subnetworks in CARP-640M. Right: Masked language modeling performance on suppression and maintenance categories of sequences for each subnetwork. (C) Dayhoff-170M-UR90. Left: Learned percent sparsity by category of learned subnetworks in Dayhoff-170M-UR90. Right: Autoregressive language modeling performance on suppression and maintenance categories of sequences for each subnetwork. Residue subnetworks were omitted from Dayhoff-170M-UR90 results because causal perplexity cannot be computed selectively over individual residues. (TIFF) [file pcbi.1013925.s004.tiff]

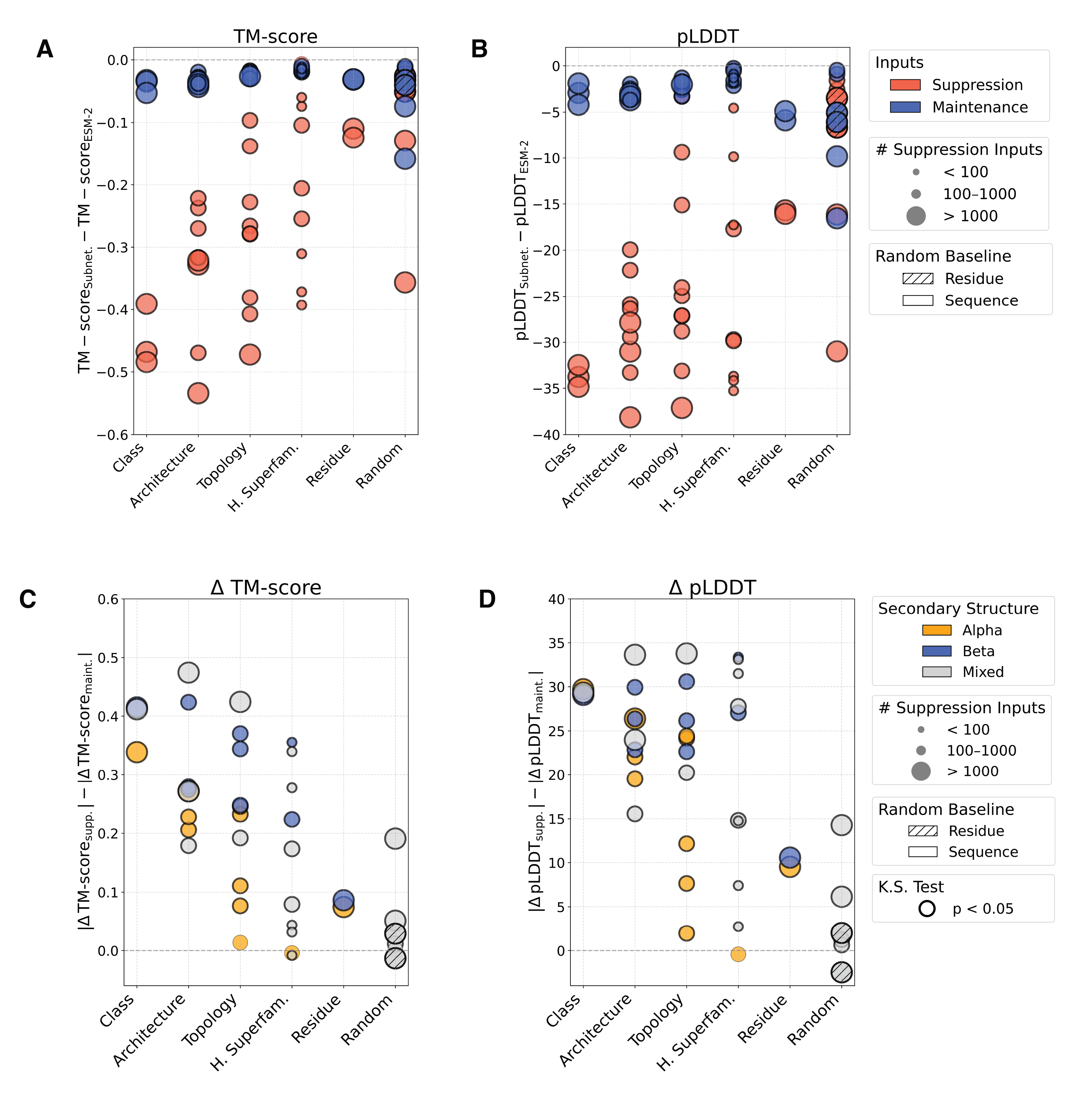

Supplement: S4 Fig — (A–B) Structural prediction differences (y-axis) between subnetworks and the ESM-2 baseline shown for (A) TM-score and (B) pLDDT across structural levels (x-axis). Each point represents change in metrics for suppression inputs (red) or maintenance inputs (blue) for a subnetwork relative to ESM-2. Marker size indicates the number of suppression inputs for each subnetwork. Bold outlines of markers indicate statistically significant paired t-test p-values (p < 0.05). (C–D) Difference in absolute structure prediction metric changes from the ESM-2 baseline (y-axis), stratified by structural level, for (C) TM-score and (D) pLDDT. Each point corresponds to an individual subnetwork and shows the difference between suppression and maintenance Δ-values. Marker size reflects the number of suppressed inputs in the subnetwork, and color indicates secondary structure type. Bold outlines of markers indicate statistical significance by Kolmogorov–Smirnov (KS) test (p < 0.05). (TIFF) [file pcbi.1013925.s005.tiff]

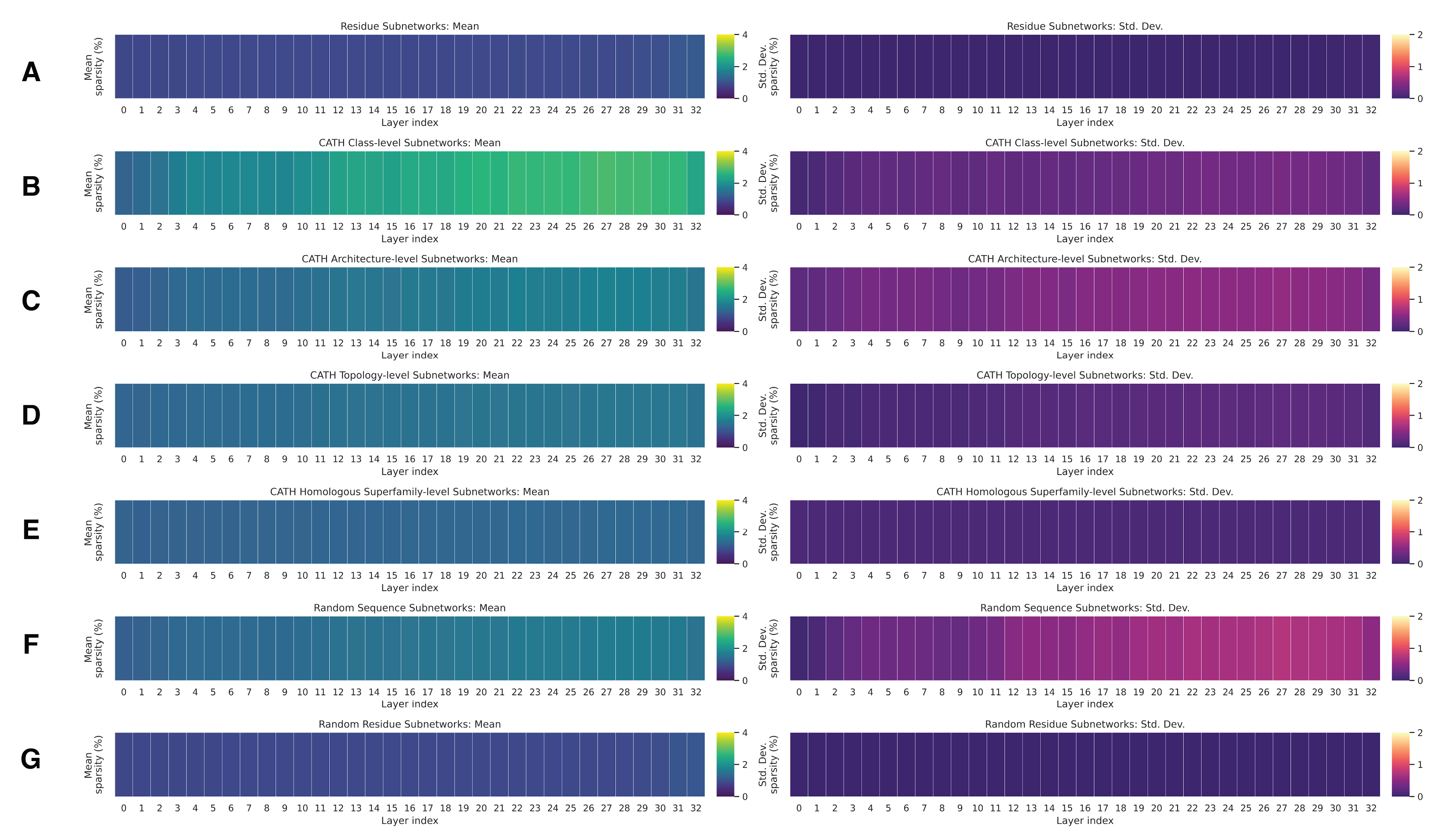

Supplement: S5 Fig — Mean and standard deviation percent of parameters pruned by layer for subnetworks grouped at the levels of (A) residue, (B) CATH class, (C) CATH architecture, (D) CATH topology, (E) CATH homologous superfamily, (F) random sequence suppression, and (G) random residue suppression. (TIFF) [file pcbi.1013925.s006.tiff]

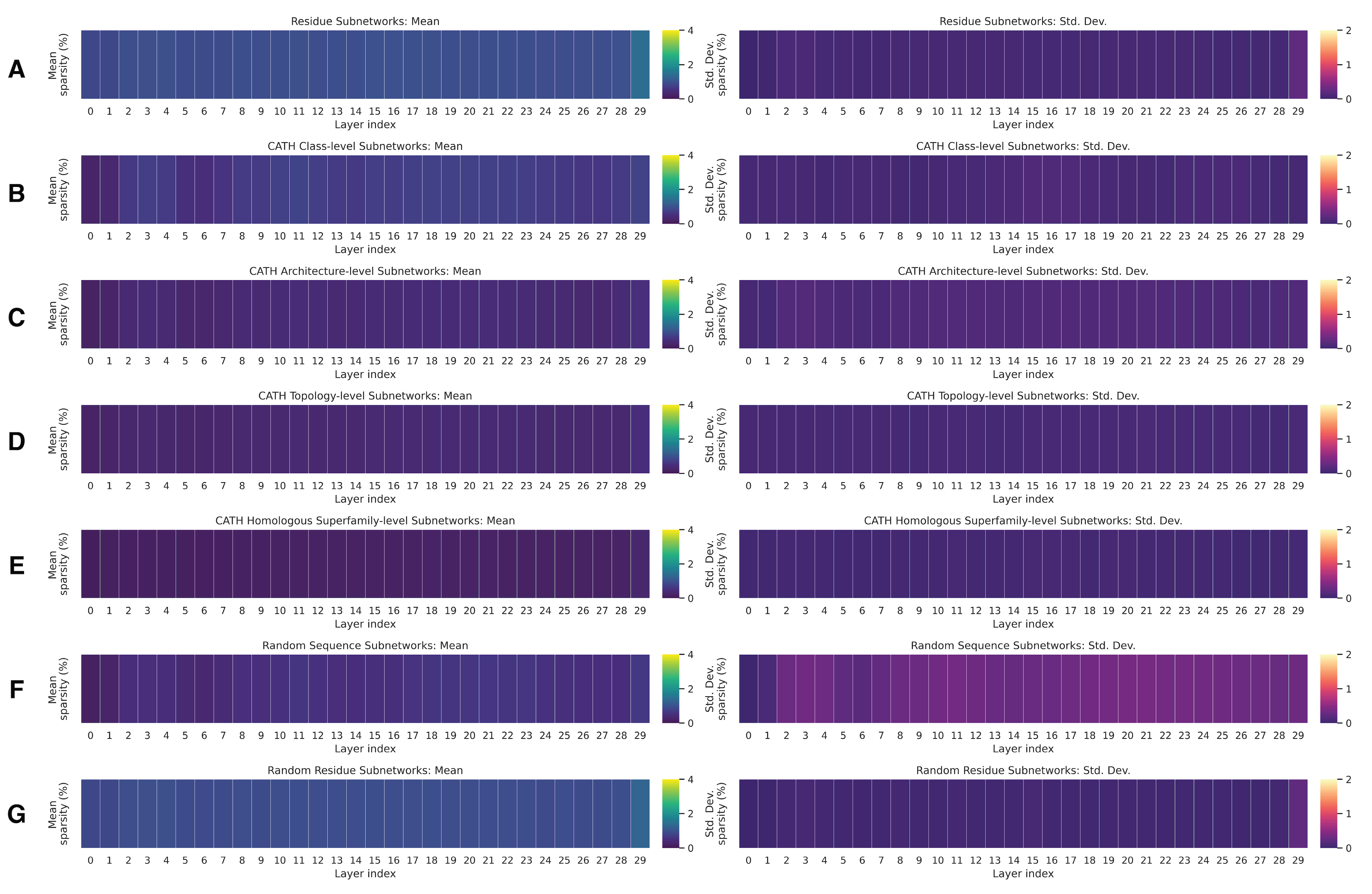

Supplement: S6 Fig — Mean and standard deviation percent of parameters pruned by layer for subnetworks grouped at the levels of (A) residue, (B) CATH class, (C) CATH architecture, (D) CATH topology, (E) CATH homologous superfamily, (F) random sequence suppression, and (G) random residue suppression. (TIFF) [file pcbi.1013925.s007.tiff]

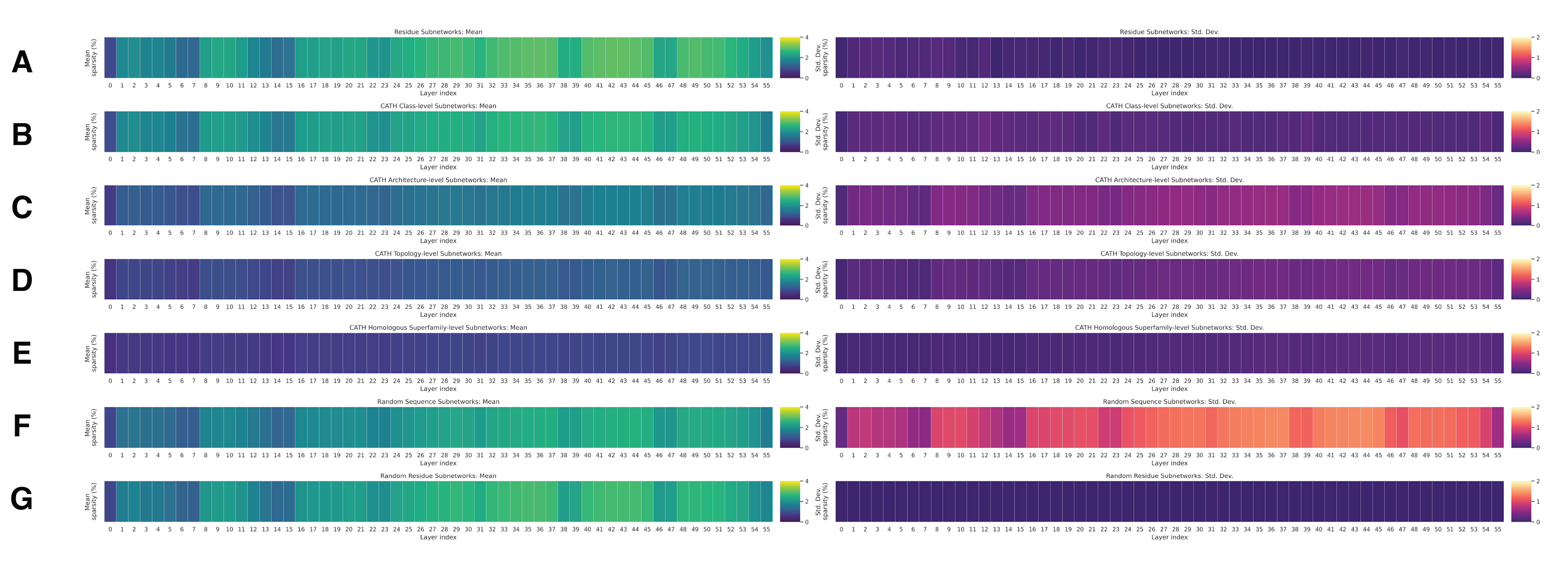

Supplement: S7 Fig — Mean and standard deviation percent of parameters pruned by layer for subnetworks grouped at the levels of (A) residue, (B) CATH class, (C) CATH architecture, (D) CATH topology, (E) CATH homologous superfamily, (F) random sequence suppression, and (G) random residue suppression. (TIFF) [file pcbi.1013925.s008.tiff]

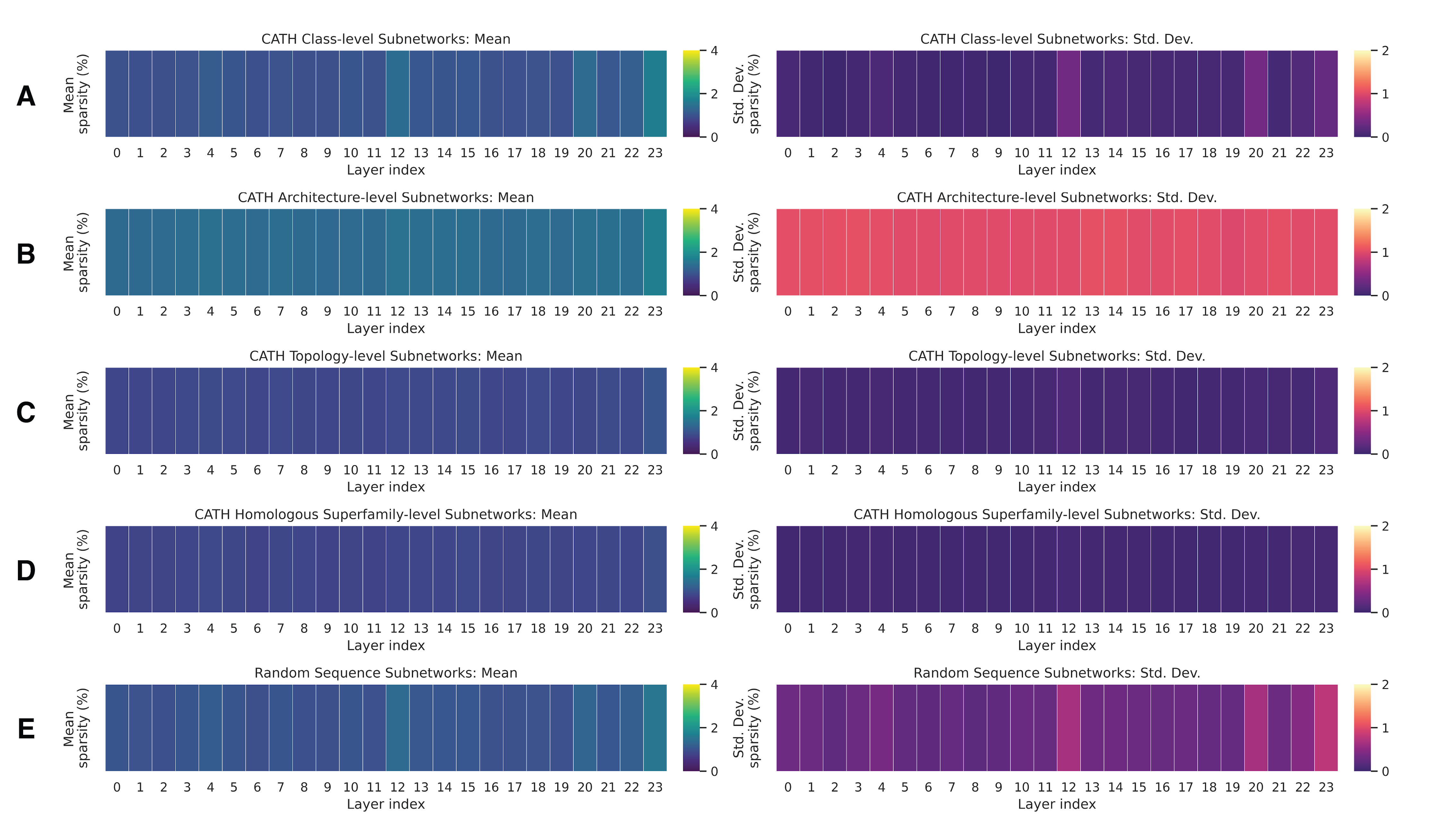

Supplement: S8 Fig — Mean and standard deviation percent of parameters pruned by layer for subnetworks grouped at the levels of (A) CATH class, (B) CATH architecture, (C) CATH topology, (D) CATH homologous superfamily, and (E) random sequence suppression. (TIFF) [file pcbi.1013925.s009.tiff]
