# Supplementary material for: Trainable subnetworks reveal insights into structure knowledge organization in protein language models
Source: PLoS Comput Biol. 2026 Feb 9;22(2):e1013925. doi: 10.1371/journal.pcbi.1013925 (PMC12928587; doi:10.1371/journal.pcbi.1013925)
Supplement: S1 Table — Each PLM subnetwork differs in which modules are masked, according to the model architecture, following evidence that knowledge is localized in representational modules [22]. Masking is thus applied to self-attention or convolutional projections while leaving embeddings, bias, and normalization layers intact. Subnetworks trained within a single PLM reuse the same hyperparameter configuration shown below. ESM-2 650M, ProtBERT-UR100, and CARP-640M are masked-language-model PLMs trained with the same MLM objective, whereas Dayhoff-170M-UR90 is an autoregressive model trained with a next-token prediction objective. (PDF) [file pcbi.1013925.s010.pdf]

S1 Table.

Model	Masked Modules	Layers	s_{init}	λ_{maint}	λ_{supp}	λ_{MLM}	τ_{init}	τ_{final}	T
ESM-2 650M	Self-attention layers	34	0.96	7	10	1	3	0.01	0.40
ProtBERT-UR100	Self-attention layers	30	0.998	9	10	1	1	0.1	0.43
CARP-640M	Convolutional kernels	56	0.995	9	10	1	1	0.1	0.40
Dayhoff-170M-UR90	Self-attention + Mamba projections	24	0.99	4	4	4	1	0.1	0.40
