# Supplementary material for: Trainable subnetworks reveal insights into structure knowledge organization in protein language models
Source: PLoS Comput Biol. 2026 Feb 9;22(2):e1013925. doi: 10.1371/journal.pcbi.1013925 (PMC12928587; doi:10.1371/journal.pcbi.1013925)
Supplement: S2 Table — Three subnetworks were independently trained for each CATH Class suppression target (Mainly Alpha, Mainly Beta, Alpha-Beta) to assess the reproducibility of mask learning given random initialization of mask scores. Reported are the mean ± standard deviation of masked language modeling perplexity for subnetworks and baseline ESM-2 perplexity stratified by categories of inputs. (PDF) [file pcbi.1013925.s011.pdf]

S2 Table.

Category	Target	Sparsity	Training Step	Subnet. Supp.	Subnet. Maint.	ESM Supp.	ESM Maint.	t-Test (p) Supp.	t-Test (p) Maint.
Class	1	2.47	7,600	24.9 ± 72.5	8.6 ± 17.4	8.6 ± 15.2	8.8 ± 14.1	$< 1e{-}16$	3.7e-05
	1	2.45	7,640	26.8 ± 82.5	8.6 ± 16.1	8.6 ± 15.2	8.8 ± 14.1	$< 1e{-}16$	2.4e-12
	1	2.48	7,560	28.3 ± 82.9	8.6 ± 16.2	8.6 ± 15.2	8.8 ± 14.1	$< 1e{-}16$	2.2e-13
	2	2.4	7,040	28.0 ± 70.2	8.7 ± 16.0	8.9 ± 15.7	8.7 ± 14.0	$< 1e{-}16$	6.5e-03
	2	2.36	7,160	25.8 ± 62.8	8.7 ± 16.3	8.9 ± 15.7	8.7 ± 14.0	$< 1e{-}16$	1.5e-02
	2	2.4	7,120	24.6 ± 59.8	8.7 ± 15.4	8.9 ± 15.7	8.7 ± 14.0	$< 1e{-}16$	3.0e-01
	3	2.56	7,880	48.3 ± 106.4	8.3 ± 22.4	8.9 ± 13.7	8.6 ± 15.2	$< 1e{-}16$	$< 1e{-}16$
	3	2.58	7,900	43.2 ± 89.9	8.1 ± 17.0	8.9 ± 13.7	8.6 ± 15.2	$< 1e{-}16$	$< 1e{-}16$
	3	2.59	8,000	38.8 ± 84.3	8.1 ± 18.0	8.9 ± 13.7	8.6 ± 15.2	$< 1e{-}16$	$< 1e{-}16$
