# Supplementary material for: Trainable subnetworks reveal insights into structure knowledge organization in protein language models
Source: PLoS Comput Biol. 2026 Feb 9;22(2):e1013925. doi: 10.1371/journal.pcbi.1013925 (PMC12928587; doi:10.1371/journal.pcbi.1013925)
Supplement: S3 Table — Each subnetwork is trained to selectively suppress either a specific type of residue belonging to a secondary structure or a set of sequences that belong to the same CATH category classification. In our study, we consider only the top 10 most frequent labels in each CATH category, and report the number of sequences and predominant type of secondary structure content in each category. (PDF) [file pcbi.1013925.s012.pdf]

S3 Table.

Category	Target	# Seqs.	Type
Residue	Helix	36.5% of residues	Alpha
	Sheet	21.9% of residues	Beta
Class	1	2169	Alpha
	2	2038	Beta
	3	3129	Mixed
Architecture	1.10	1247	Alpha
	1.25	182	Alpha
	1.20	693	Alpha
	2.30	251	Beta
	2.40	447	Beta
	2.60	896	Beta
	3.30	1396	Mixed
	3.40	1797	Mixed
	40	469	Mixed
Topology	1.10.10	192	Alpha
	1.10.287	111	Alpha
	1.20.58	135	Alpha
	1.20.120	118	Alpha
	1.25.40	142	Alpha
	2.40.50	136	Beta
	2.60.40	512	Beta
	2.60.120	276	Beta
	3.30.70	282	Mixed
	3.40.50	1110	Mixed
H. Superfam.	3.40.50.300	168	Mixed
	2.60.40.10	160	Beta
	3.40.50.720	117	Mixed
	1.10.10.10	102	Alpha
	3.20.20.80	88	Mixed
	3.40.190.10	81	Mixed
	3.40.50.150	77	Mixed
	3.40.50.1820	75	Mixed
	2.40.50.140	68	Beta
	3.40.30.10	67	Mixed
Random	Sequences	100	Mixed
		200	Mixed
		1000	Mixed
		2000	Mixed
Random	Residue	Random	Mixed
