# Supplementary material for: Trainable subnetworks reveal insights into structure knowledge organization in protein language models
Source: PLoS Comput Biol. 2026 Feb 9;22(2):e1013925. doi: 10.1371/journal.pcbi.1013925 (PMC12928587; doi:10.1371/journal.pcbi.1013925)
Supplement: S4 Table — Below we report the mean and standard deviation of the subnetwork and ESM-2 baseline perplexities (illustrated in Fig 2C) stratified by categories of inputs. The t-test is performed on paired inputs of the subnetwork performance and ESM-2 baseline performance and p-values are reported below. The random residue control subnetwork is trained to suppress random residues, but we independently evaluate and report the MLM performance of this subnetwork on alpha helices and beta sheets. All per-sequence metrics are available in CSVs in the code repository. (PDF) [file pcbi.1013925.s013.pdf]

S4 Table.

Category	Target	Sparsity	Training Step	Subnet. Supp.	Subnet. Maint.	ESM-2 Supp.	ESM-2 Maint.	t-Test (p) Supp.	t-Test (p) Maint.
Residue	Helix	0.90	784	7.9 ± 5.0	5.0 ± 5.2	5.6 ± 4.0	4.9 ± 5.1	$< 1e{-16}$	$< 1e{-16}$
	Sheet	0.89	784	6.7 ± 6.1	5.6 ± 4.1	4.9 ± 5.1	5.6 ± 4.0	$< 1e{-16}$	$< 1e{-16}$
Class	1	2.13	7,680	27.2 ± 80.3	8.0 ± 15.4	8.6 ± 15.2	8.8 ± 14.1	$< 1e{-16}$	$< 1e{-16}$
	2	2.06	7,200	28.7 ± 74.1	8.2 ± 15.5	8.9 ± 15.7	8.7 ± 14.0	$< 1e{-16}$	$< 1e{-16}$
	3	2.53	7,820	41.5 ± 91.4	8.2 ± 18.5	8.9 ± 13.7	8.6 ± 15.2	$< 1e{-16}$	$< 1e{-16}$
Architecture	1.10	2.02	4,440	11.0 ± 21.7	9.3 ± 16.4	7.6 ± 15.2	8.9 ± 14.3	$< 1e{-16}$	$< 1e{-16}$
	1.20	1.25	3,660	14.1 ± 28.0	9.3 ± 17.6	9.0 ± 12.4	8.7 ± 14.5	$< 1e{-16}$	$< 1e{-16}$
	1.25	1.31	640	34.9 ± 70.6	9.1 ± 16.8	11.9 ± 19.6	8.7 ± 14.2	$< 1e{-16}$	$< 1e{-16}$
	2.30	1.05	1,680	9.9 ± 12.7	9.5 ± 17.0	5.1 ± 5.1	8.8 ± 14.5	$< 1e{-16}$	$< 1e{-16}$
	2.40	1.10	2,280	13.0 ± 21.4	9.3 ± 17.1	7.2 ± 10.5	8.8 ± 14.6	$< 1e{-16}$	$< 1e{-16}$
	2.60	1.71	3,000	20.0 ± 37.6	9.2 ± 16.5	8.9 ± 14.1	8.7 ± 14.4	$< 1e{-16}$	$< 1e{-16}$
	3.30	1.68	4,880	10.1 ± 14.6	9.3 ± 17.6	7.3 ± 10.6	9.0 ± 15.0	$< 1e{-16}$	$< 1e{-16}$
	3.40	2.18	6,200	34.9 ± 67.5	8.8 ± 18.2	9.2 ± 14.0	8.6 ± 14.5	$< 1e{-16}$	$< 1e{-16}$
	3.90	1.41	1,640	14.9 ± 28.0	9.0 ± 16.3	10.8 ± 19.4	8.6 ± 14.1	$< 1e{-16}$	$< 1e{-16}$
Topology	1.10.10	1.51	1,280	6.9 ± 9.6	9.7 ± 17.5	4.6 ± 6.7	8.8 ± 14.5	$< 1e{-16}$	$< 1e{-16}$
	1.10.287	1.37	720	5.3 ± 5.7	9.4 ± 17.1	5.2 ± 6.1	8.8 ± 14.5	$4.5e-01$	$< 1e{-16}$
	1.20.120	1.38	800	11.9 ± 14.5	9.5 ± 16.9	8.3 ± 8.5	8.7 ± 14.5	$< 1e{-16}$	$< 1e{-16}$
	1.20.58	1.41	960	13.4 ± 19.8	9.6 ± 17.3	8.4 ± 10.8	8.7 ± 14.4	$< 1e{-16}$	$< 1e{-16}$
	1.25.40	1.34	780	40.8 ± 94.2	9.2 ± 16.7	11.0 ± 19.2	8.7 ± 14.3	$< 1e{-16}$	$< 1e{-16}$
	2.40.50	1.36	720	8.7 ± 9.7	9.3 ± 16.4	5.2 ± 5.8	8.8 ± 14.5	$< 1e{-16}$	$< 1e{-16}$
	2.60.120	1.37	1,000	32.7 ± 49.6	9.1 ± 17.9	11.1 ± 15.0	8.7 ± 14.4	$< 1e{-16}$	$< 1e{-16}$
	2.60.40	1.52	1,800	11.9 ± 15.3	9.4 ± 17.5	7.1 ± 9.7	8.8 ± 14.6	$< 1e{-16}$	$< 1e{-16}$
	3.30.70	1.52	1,500	8.3 ± 9.4	9.6 ± 18.1	5.7 ± 6.3	8.8 ± 14.6	$< 1e{-16}$	$< 1e{-16}$
	3.40.50	1.80	3,840	25.6 ± 40.2	9.1 ± 17.7	8.8 ± 11.1	8.7 ± 14.8	$< 1e{-16}$	$< 1e{-16}$
H. Superfam.	1.10.10.10	1.26	300	4.0 ± 3.4	9.0 ± 16.5	4.1 ± 3.9	8.8 ± 14.5	$4.1e-02$	$< 1e{-16}$
	2.40.50.140	1.38	480	10.2 ± 11.0	9.5 ± 17.4	4.6 ± 3.6	8.8 ± 14.4	$< 1e{-16}$	$< 1e{-16}$
	2.60.40.10	1.04	840	8.8 ± 9.1	9.2 ± 16.4	4.9 ± 4.3	8.8 ± 14.5	$< 1e{-16}$	$< 1e{-16}$
	3.20.20.80	1.26	360	46.4 ± 55.6	9.1 ± 16.8	12.9 ± 14.5	8.7 ± 14.4	$< 1e{-16}$	$< 1e{-16}$
	3.40.190.10	1.29	420	22.7 ± 30.1	9.2 ± 16.5	8.9 ± 8.6	8.7 ± 14.4	$< 1e{-16}$	$< 1e{-16}$
	3.40.30.10	1.32	480	15.1 ± 13.5	9.3 ± 17.5	5.1 ± 4.4	8.8 ± 14.4	$< 1e{-16}$	$< 1e{-16}$
	3.40.50.150	1.31	480	17.5 ± 24.0	9.2 ± 17.5	9.5 ± 12.1	8.7 ± 14.4	$< 1e{-16}$	$< 1e{-16}$
	3.40.50.1820	1.27	360	20.2 ± 21.8	9.1 ± 16.5	12.2 ± 12.6	8.7 ± 14.4	$< 1e{-16}$	$< 1e{-16}$
	3.40.50.300	1.30	560	21.6 ± 28.1	9.1 ± 16.3	9.2 ± 10.2	8.7 ± 14.5	$< 1e{-16}$	$< 1e{-16}$
	3.40.50.720	1.32	660	27.6 ± 49.2	9.2 ± 16.7	7.4 ± 7.8	8.8 ± 14.5	$< 1e{-16}$	$< 1e{-16}$
Random	Helix	0.87	196	6.2 ± 4.3	5.2 ± 5.2	5.6 ± 4.0	4.9 ± 5.1	$< 1e{-16}$	$< 1e{-16}$
	Sheet	0.87	196	5.2 ± 5.2	6.2 ± 4.3	4.9 ± 5.1	5.6 ± 4.0	$< 1e{-16}$	$< 1e{-16}$
	100 seqs.	1.17	420	9.2 ± 13.2	8.9 ± 15.7	8.4 ± 12.0	8.7 ± 14.4	$1.8e-09$	$< 1e{-16}$
	200 seqs.	1.21	780	9.2 ± 13.2	9.0 ± 15.7	8.3 ± 11.8	8.7 ± 14.4	$< 1e{-16}$	$< 1e{-16}$
	1000 seqs.	1.37	2,520	8.5 ± 13.4	8.9 ± 16.2	7.8 ± 11.5	8.8 ± 14.6	$< 1e{-16}$	$2.1e-04$
	2000 seqs.	2.17	7,500	11.2 ± 21.3	8.2 ± 15.6	8.6 ± 14.0	8.8 ± 14.5	$< 1e{-16}$	$< 1e{-16}$
