# Supplementary material for: Trainable subnetworks reveal insights into structure knowledge organization in protein language models
Source: PLoS Comput Biol. 2026 Feb 9;22(2):e1013925. doi: 10.1371/journal.pcbi.1013925 (PMC12928587; doi:10.1371/journal.pcbi.1013925)
Supplement: S5 Table — Reported values correspond to mean, standard deviation, minimum, and maximum perplexities across all CATH domains within each structural CATH Class. (PDF) [file pcbi.1013925.s014.pdf]

S5 Table.

Category	Count (n)	Mean	Std. Dev.	Min	Max
Mainly Alpha	10506	4.49	3.02	1.51	44.00
Mainly Beta	8493	3.71	2.15	1.56	35.19
Alpha-Beta	19092	3.50	1.97	1.65	57.61
