# Supplementary material for: Trainable subnetworks reveal insights into structure knowledge organization in protein language models
Source: PLoS Comput Biol. 2026 Feb 9;22(2):e1013925. doi: 10.1371/journal.pcbi.1013925 (PMC12928587; doi:10.1371/journal.pcbi.1013925)
Supplement: S6 Table — For TM-score, RMSD, and pLDDT, we report the mean ± standard deviation of the subnetwork performance across all sequences within each category of suppression and maintenance inputs. ESM-2 (650M) performance on the same categories is reported as the PLM baseline. To quantify the differences in subnetwork and PLM performance, we perform a paired t-test on all (i) suppression inputs and (ii) maintenance inputs, computing the difference in Δmetric=metricSubnet.−metricESM-2.. We then perform a Kolmogorov–Smirnov (KS) test on these differences to assess whether the distribution of |Δmetric,supp| is significantly greater than that of |Δmetric,maint|. Our evaluation scheme is illustrated in Fig 3A. We report p-values for both statistical tests for each subnetwork; significant p-values are in bold. For residue-level suppression, we evaluate structure prediction on CATH class categories of mainly alpha and mainly beta sequences as a proxy for evaluating alpha helix and beta strand performance. We report performance on the only residue-specific metric of pLDDT in Fig 3F and 3G. The random residue suppression subnetwork, i.e. residue-control, is one subnetwork but we evaluated it separately on alpha helices and beta sheets. All per-sequence structure prediction metrics are provided via CSVs in our code repository. (PDF) [file pcbi.1013925.s015.pdf]

S6 Table.

**Category**	**Target**	**Metric**	**Subnet. Supp.**	**Subnet. Maint.**	**ESM-2 Supp.**	**ESM-2 Maint.**	**t-Test (p) Supp.**	**t-Test (p) Maint.**	**K.S.-test (p)**
Residue	Helix	RMSD	3.62 ± 1.19	3.05 ± 1.09	2.93 ± 1.07	2.83 ± 1.09	**< 1e−16**	**< 1e−16**	**< 1e−16**
		TM-score	0.55 ± 0.20	0.68 ± 0.19	0.66 ± 0.20	0.71 ± 0.19	**< 1e−16**	**< 1e−16**	**< 1e−16**
		pLDDT	50.81±13.89	59.49±12.79	66.51±12.82	65.38±12.69	**< 1e−16**	**< 1e−16**	**< 1e−16**
	Sheet	RMSD	3.74 ± 1.43	3.05 ± 1.12	2.89 ± 1.19	2.85 ± 1.11	**< 1e−16**	**< 1e−16**	**< 1e−16**
		TM-score	0.57 ± 0.21	0.66 ± 0.20	0.70 ± 0.19	0.69 ± 0.20	**< 1e−16**	**< 1e−16**	**< 1e−16**
		pLDDT	47.79±13.55	60.98±12.72	63.86±13.49	65.87±12.65	**< 1e−16**	**< 1e−16**	**< 1e−16**
Class	1	RMSD	5.22 ± 1.42	3.05 ± 1.10	2.86 ± 1.12	2.82 ± 1.10	**< 1e−16**	**< 1e−16**	**< 1e−16**
		TM-score	0.27 ± 0.12	0.68 ± 0.19	0.66 ± 0.21	0.71 ± 0.20	**< 1e−16**	**< 1e−16**	**< 1e−16**
		pLDDT	33.31 ± 8.37	62.30±13.07	67.09±13.15	65.24±12.92	**< 1e−16**	**< 1e−16**	**< 1e−16**
	2	RMSD	6.02 ± 1.28	3.08 ± 1.10	2.93 ± 1.15	2.84 ± 1.09	**< 1e−16**	**< 1e−16**	**< 1e−16**
		TM-score	0.23 ± 0.11	0.66 ± 0.20	0.69 ± 0.19	0.69 ± 0.20	**< 1e−16**	**< 1e−16**	**< 1e−16**
		pLDDT	30.53 ± 7.66	64.25±12.91	63.02±13.59	66.16±12.66	**< 1e−16**	**< 1e−16**	**< 1e−16**
	3	RMSD	6.13 ± 1.39	3.26 ± 1.15	2.81 ± 1.06	2.93 ± 1.13	**< 1e−16**	**< 1e−16**	**< 1e−16**
		TM-score	0.23 ± 0.12	0.61 ± 0.20	0.72 ± 0.19	0.67 ± 0.21	**< 1e−16**	**< 1e−16**	**< 1e−16**
		pLDDT	30.80 ± 7.97	60.54±13.99	65.64±12.55	64.75±13.30	**< 1e−16**	**< 1e−16**	**< 1e−16**
Arch.	1.10	RMSD	4.80 ± 1.20	3.12 ± 1.16	2.85 ± 1.13	2.83 ± 1.11	**< 1e−16**	**< 1e−16**	**< 1e−16**
		TM-score	0.32 ± 0.13	0.66 ± 0.21	0.65 ± 0.20	0.71 ± 0.20	**< 1e−16**	**< 1e−16**	**< 1e−16**
		pLDDT	35.91 ± 8.20	61.82±14.58	66.94±12.45	65.53±12.96	**< 1e−16**	**< 1e−16**	**< 1e−16**
	1.20	RMSD	4.59 ± 1.27	3.05 ± 1.16	3.06 ± 1.08	2.84 ± 1.10	**< 1e−16**	**< 1e−16**	**< 1e−16**
		TM-score	0.36 ± 0.17	0.66 ± 0.21	0.63 ± 0.20	0.70 ± 0.20	**< 1e−16**	**< 1e−16**	**< 1e−16**
		pLDDT	39.83±13.38	62.42±14.80	65.69±13.68	65.36±13.01	**< 1e−16**	**< 1e−16**	**< 1e−16**
	1.25	RMSD	4.29 ± 1.40	3.00 ± 1.10	2.67 ± 1.04	2.86 ± 1.10	**4.6e-12**	**< 1e−16**	**< 1e−16**
		TM-score	0.54 ± 0.22	0.67 ± 0.20	0.78 ± 0.15	0.69 ± 0.20	**7.5e-12**	**< 1e−16**	**< 1e−16**
		pLDDT	49.23±15.56	63.32±13.03	71.39±11.65	65.30±12.65	**5.7e-16**	**< 1e−16**	**< 1e−16**
	2.30	RMSD	4.66 ± 1.21	3.06 ± 1.17	2.69 ± 0.95	2.86 ± 1.12	**< 1e−16**	**< 1e−16**	**< 1e−16**
		TM-score	0.37 ± 0.18	0.67 ± 0.20	0.68 ± 0.18	0.70 ± 0.20	**< 1e−16**	**< 1e−16**	**< 1e−16**
		pLDDT	37.72±12.77	62.99±14.17	67.14±13.07	65.61±13.01	**< 1e−16**	**< 1e−16**	**< 1e−16**
	2.40	RMSD	5.12 ± 1.45	3.00 ± 1.15	2.99 ± 1.07	2.81 ± 1.09	**< 1e−16**	**< 1e−16**	**< 1e−16**
		TM-score	0.34 ± 0.20	0.67 ± 0.20	0.66 ± 0.19	0.70 ± 0.20	**< 1e−16**	**< 1e−16**	**< 1e−16**
		pLDDT	35.83±12.39	63.38±13.86	62.20±14.02	66.23±12.59	**< 1e−16**	**< 1e−16**	**< 1e−16**
	2.60	RMSD	6.03 ± 1.09	3.07 ± 1.15	2.84 ± 1.16	2.86 ± 1.11	**< 1e−16**	**< 1e−16**	**< 1e−16**
		TM-score	0.24 ± 0.12	0.66 ± 0.21	0.71 ± 0.18	0.69 ± 0.20	**< 1e−16**	**< 1e−16**	**< 1e−16**
		pLDDT	29.96 ± 6.60	63.17±13.73	63.26±13.72	65.66±12.94	**< 1e−16**	**< 1e−16**	**< 1e−16**
	3.30	RMSD	4.93 ± 1.24	3.05 ± 1.14	2.87 ± 1.14	2.80 ± 1.09	**< 1e−16**	**< 1e−16**	**< 1e−16**
		TM-score	0.34 ± 0.16	0.67 ± 0.20	0.66 ± 0.20	0.71 ± 0.20	**< 1e−16**	**< 1e−16**	**< 1e−16**
		pLDDT	36.39±11.38	63.25±13.87	64.26±14.23	66.24±12.61	**< 1e−16**	**< 1e−16**	**< 1e−16**
	3.40	RMSD	6.42 ± 1.24	3.18 ± 1.14	2.60 ± 0.98	2.92 ± 1.13	**< 1e−16**	**< 1e−16**	**< 1e−16**
		TM-score	0.24 ± 0.11	0.63 ± 0.20	0.77 ± 0.16	0.67 ± 0.20	**< 1e−16**	**< 1e−16**	**< 1e−16**
		pLDDT	30.33 ± 6.87	61.31±13.65	68.47±10.68	64.63±13.30	**< 1e−16**	**< 1e−16**	**< 1e−16**
	3.90	RMSD	4.85 ± 1.49	3.10 ± 1.22	3.32 ± 1.38	2.84 ± 1.11	**< 1e−16**	**< 1e−16**	**< 1e−16**
		TM-score	0.42 ± 0.21	0.66 ± 0.21	0.65 ± 0.23	0.70 ± 0.20	**< 1e−16**	**< 1e−16**	**< 1e−16**
		pLDDT	41.17±12.83	61.81±15.13	61.12±13.68	65.50±13.06	**< 1e−16**	**< 1e−16**	**< 1e−16**
Topo.	1.10.10	RMSD	3.90 ± 1.10	3.05 ± 1.15	2.35 ± 0.90	2.87 ± 1.13	**9.0e-14**	**< 1e−16**	**< 1e−16**
		TM-score	0.41 ± 0.16	0.67 ± 0.20	0.68 ± 0.18	0.70 ± 0.20	**1.3e-14**	**< 1e−16**	**< 1e−16**
		pLDDT	44.81±14.34	62.72±14.01	71.96±10.84	65.30±13.04	**< 1e−16**	**< 1e−16**	**< 1e−16**
	1.10.287	RMSD	2.80 ± 0.93	2.98 ± 1.08	2.77 ± 0.86	2.85 ± 1.08	7.6e-01	**< 1e−16**	**3.2e-02**
		TM-score	0.56 ± 0.17	0.68 ± 0.19	0.59 ± 0.18	0.70 ± 0.20	**6.2e-03**	**< 1e−16**	6.1e-02
		pLDDT	67.32±13.60	63.70±12.75	70.62±11.32	65.74±12.57	**5.9e-03**	**< 1e−16**	**4.6e-02**
	1.20.120	RMSD	4.10 ± 1.35	3.00 ± 1.11	3.28 ± 1.13	2.83 ± 1.09	**1.4e-04**	**< 1e−16**	**2.8e-05**
		TM-score	0.47 ± 0.19	0.67 ± 0.20	0.61 ± 0.20	0.70 ± 0.20	**7.8e-07**	**< 1e−16**	**9.6e-11**
		pLDDT	48.32±14.04	63.08±13.69	63.44±12.66	65.64±12.96	**1.6e-07**	**< 1e−16**	**1.2e-10**
	1.20.58	RMSD	3.40 ± 0.98	3.00 ± 1.10	2.93 ± 1.13	2.85 ± 1.10	**3.3e-04**	**< 1e−16**	**4.3e-04**
		TM-score	0.50 ± 0.16	0.68 ± 0.20	0.60 ± 0.21	0.70 ± 0.20	**1.4e-05**	**< 1e−16**	**5.6e-08**

Category	Target	Metric	Subnet. Supp.	Subnet. Maint.	ESM-2 Supp.	ESM-2 Maint.	t-Test (p) Supp.	t-Test (p) Maint.	K.S.-test (p)
		pLDDT	55.58±12.59	63.37±13.01	64.98±14.31	65.58±12.65	**7.5e-06**	**< 1e−16**	**5.6e-07**
	1.25.40	RMSD	4.21 ± 1.60	2.98 ± 1.13	2.65 ± 1.14	2.85 ± 1.11	**1.3e-08**	**< 1e−16**	**1.0e-12**
		TM-score	0.49 ± 0.25	0.68 ± 0.20	0.77 ± 0.15	0.70 ± 0.20	**5.2e-10**	**< 1e−16**	**2.1e-16**
		pLDDT	47.61±17.00	63.50±13.65	74.72±10.08	65.48±13.00	**1.7e-13**	**< 1e−16**	**< 1e−16**
	2.40.50	RMSD	4.66 ± 1.19	2.98 ± 1.12	2.86 ± 1.11	2.86 ± 1.10	**1.1e-11**	**< 1e−16**	**< 1e−16**
		TM-score	0.35 ± 0.17	0.68 ± 0.20	0.63 ± 0.21	0.69 ± 0.20	**6.2e-11**	**< 1e−16**	**< 1e−16**
		pLDDT	37.35±10.71	63.53±13.34	62.32±14.64	65.39±12.84	**2.3e-12**	**< 1e−16**	**< 1e−16**
	2.60.120	RMSD	5.75 ± 1.65	3.00 ± 1.15	2.91 ± 1.15	2.86 ± 1.12	**< 1e−16**	**< 1e−16**	**< 1e−16**
		TM-score	0.36 ± 0.22	0.67 ± 0.21	0.74 ± 0.17	0.69 ± 0.20	**< 1e−16**	**< 1e−16**	**< 1e−16**
		pLDDT	33.70±10.54	63.74±13.81	62.51±12.81	65.47±13.04	**< 1e−16**	**< 1e−16**	**< 1e−16**
	2.60.40	RMSD	5.29 ± 1.24	3.02 ± 1.14	2.61 ± 0.91	2.85 ± 1.10	**< 1e−16**	**< 1e−16**	**< 1e−16**
		TM-score	0.32 ± 0.17	0.67 ± 0.20	0.73 ± 0.15	0.69 ± 0.20	**< 1e−16**	**< 1e−16**	**< 1e−16**
		pLDDT	32.83 ± 8.78	64.14±13.58	65.96±12.34	65.44±12.82	**< 1e−16**	**< 1e−16**	**< 1e−16**
	3.30.70	RMSD	4.12 ± 1.11	3.05 ± 1.14	2.72 ± 1.13	2.84 ± 1.11	**< 1e−16**	**< 1e−16**	**< 1e−16**
		TM-score	0.44 ± 0.18	0.67 ± 0.20	0.67 ± 0.20	0.70 ± 0.20	**< 1e−16**	**< 1e−16**	**< 1e−16**
		pLDDT	41.78±13.24	62.50±13.90	65.82±14.80	65.76±12.80	**< 1e−16**	**< 1e−16**	**< 1e−16**
	3.40.50	RMSD	5.99 ± 1.16	3.09 ± 1.13	2.53 ± 0.88	2.91 ± 1.14	**< 1e−16**	**< 1e−16**	**< 1e−16**
		TM-score	0.32 ± 0.14	0.66 ± 0.20	0.80 ± 0.14	0.68 ± 0.20	**< 1e−16**	**< 1e−16**	**< 1e−16**
		pLDDT	32.65 ± 6.56	62.69±13.36	69.77 ± 9.16	64.73±13.38	**< 1e−16**	**< 1e−16**	**< 1e−16**
H. Supfam.	1.10.10.10	RMSD	2.30 ± 0.99	2.92 ± 1.10	2.28 ± 0.89	2.83 ± 1.09	8.8e-01	**< 1e−16**	1.8e-01
		TM-score	0.71 ± 0.17	0.69 ± 0.20	0.72 ± 0.16	0.70 ± 0.20	4.7e-01	**< 1e−16**	7.0e-02
		pLDDT	72.62±11.95	65.18±12.92	73.16±11.74	65.74±12.81	9.3e-02	**< 1e−16**	4.5e-01
	2.40.50.140	RMSD	5.03 ± 0.96	2.99 ± 1.09	2.49 ± 0.90	2.85 ± 1.08	**1.1e-08**	**< 1e−16**	**< 1e−16**
		TM-score	0.32 ± 0.14	0.68 ± 0.20	0.72 ± 0.17	0.70 ± 0.20	**6.3e-08**	**< 1e−16**	**2.4e-14**
		pLDDT	34.61 ± 8.13	63.92±13.16	68.78±12.37	65.73±12.64	**1.1e-07**	**< 1e−16**	**< 1e−16**
	2.60.40.10	RMSD	3.75 ± 1.26	3.03 ± 1.14	2.17 ± 0.69	2.89 ± 1.14	**7.2e-13**	**< 1e−16**	**< 1e−16**
		TM-score	0.53 ± 0.19	0.67 ± 0.20	0.79 ± 0.10	0.69 ± 0.20	**5.4e-15**	**< 1e−16**	**< 1e−16**
		pLDDT	41.57±12.07	63.52±13.34	71.28 ± 9.64	65.13±13.02	**< 1e−16**	**< 1e−16**	**< 1e−16**
	3.20.20.80	RMSD	3.45 ± 0.94	2.96 ± 1.11	2.63 ± 0.66	2.86 ± 1.10	**6.4e-06**	**< 1e−16**	**1.8e-07**
		TM-score	0.79 ± 0.10	0.68 ± 0.20	0.87 ± 0.07	0.69 ± 0.20	**1.2e-05**	**< 1e−16**	**6.3e-08**
		pLDDT	49.76 ± 9.16	63.98±13.30	67.03 ± 7.20	65.46±12.91	**8.5e-10**	**< 1e−16**	**< 1e−16**
	3.40.190.10	RMSD	5.22 ± 1.21	3.00 ± 1.14	2.84 ± 1.06	2.89 ± 1.15	**5.1e-11**	**< 1e−16**	**< 1e−16**
		TM-score	0.32 ± 0.11	0.67 ± 0.20	0.63 ± 0.12	0.69 ± 0.20	**2.6e-09**	**< 1e−16**	**< 1e−16**
		pLDDT	32.96 ± 5.12	64.06±13.44	66.66 ± 6.61	65.12±13.23	**9.4e-16**	**< 1e−16**	**< 1e−16**
	3.40.30.10	RMSD	4.49 ± 1.63	2.95 ± 1.09	1.75 ± 0.48	2.86 ± 1.11	**1.7e-06**	**< 1e−16**	**1.2e-14**
		TM-score	0.46 ± 0.21	0.68 ± 0.20	0.84 ± 0.08	0.69 ± 0.20	**1.7e-06**	**< 1e−16**	**2.5e-15**
		pLDDT	41.38±14.66	64.21±12.76	76.66 ± 7.22	65.51±12.75	**1.7e-09**	**< 1e−16**	**< 1e−16**
	3.40.50.150	RMSD	2.85 ± 1.23	2.94 ± 1.11	2.40 ± 0.77	2.84 ± 1.10	**2.2e-03**	**< 1e−16**	**1.5e-03**
		TM-score	0.79 ± 0.16	0.68 ± 0.20	0.85 ± 0.10	0.70 ± 0.20	**5.8e-04**	**< 1e−16**	**2.8e-04**
		pLDDT	62.15±12.19	63.90±12.84	72.03 ± 9.41	65.70±12.82	**8.7e-10**	**< 1e−16**	**< 1e−16**
	3.40.50.1820	RMSD	2.57 ± 0.70	2.95 ± 1.13	2.39 ± 0.68	2.87 ± 1.12	**4.9e-05**	**< 1e−16**	**1.7e-02**
		TM-score	0.86 ± 0.07	0.68 ± 0.20	0.88 ± 0.06	0.69 ± 0.20	**2.9e-06**	**< 1e−16**	**2.5e-03**
		pLDDT	66.94 ± 8.82	64.39±13.30	71.50 ± 6.95	65.29±13.09	**7.4e-08**	**< 1e−16**	**8.1e-10**
	3.40.50.300	RMSD	3.44 ± 1.11	2.99 ± 1.12	2.63 ± 0.76	2.87 ± 1.12	**2.0e-08**	**< 1e−16**	**1.7e-10**
		TM-score	0.68 ± 0.15	0.67 ± 0.20	0.79 ± 0.13	0.69 ± 0.20	**2.2e-08**	**< 1e−16**	**3.9e-12**
		pLDDT	50.62±12.41	63.22±13.53	68.34 ± 8.00	65.36±13.26	**< 1e−16**	**< 1e−16**	**< 1e−16**
	3.40.50.720	RMSD	4.26 ± 1.41	2.98 ± 1.11	2.47 ± 0.93	2.87 ± 1.11	**3.5e-08**	**< 1e−16**	**< 1e−16**
		TM-score	0.56 ± 0.19	0.68 ± 0.20	0.77 ± 0.16	0.70 ± 0.20	**5.4e-09**	**< 1e−16**	**< 1e−16**
		pLDDT	42.35±11.23	65.14±13.51	72.20±10.85	65.42±12.87	**1.2e-14**	**1.3e-04**	**< 1e−16**
Random	100 seqs.	RMSD	3.06 ± 1.17	2.92 ± 1.11	2.92 ± 1.17	2.84 ± 1.11	**1.0e-03**	**< 1e−16**	5.2e-01
		TM-score	0.66 ± 0.20	0.69 ± 0.20	0.67 ± 0.21	0.70 ± 0.20	**3.7e-04**	**< 1e−16**	5.8e-01
		pLDDT	63.15±12.98	65.23±13.21	64.74±12.34	65.71±12.95	**3.4e-07**	**< 1e−16**	**5.3e-06**
	1000 seqs.	RMSD	3.64 ± 1.24	3.37 ± 1.25	2.81 ± 1.05	2.86 ± 1.12	**< 1e−16**	**< 1e−16**	**< 1e−16**
		TM-score	0.57 ± 0.21	0.62 ± 0.21	0.70 ± 0.19	0.69 ± 0.20	**< 1e−16**	**< 1e−16**	**< 1e−16**
		pLDDT	49.65±13.98	55.49±14.77	65.79±12.85	65.32±13.06	**< 1e−16**	**< 1e−16**	**< 1e−16**

**Category**	**Target**	**Metric**	**Subnet. Supp.**	**Subnet. Maint.**	**ESM-2 Supp.**	**ESM-2 Maint.**	**t-Test (p) Supp.**	**t-Test (p) Maint.**	**K.S.-test (p)**
	200 seqs.	RMSD	3.00 ± 1.16	2.95 ± 1.13	2.79 ± 1.12	2.85 ± 1.13	**4.6e-10**	$<$ **1e−16**	**1.5e-03**
		TM-score	0.67 ± 0.20	0.68 ± 0.20	0.70 ± 0.20	0.70 ± 0.20	**2.4e-12**	$<$ **1e−16**	**1.6e-04**
		pLDDT	63.04 ± 14.38	64.62 ± 13.40	65.62 ± 13.21	65.50 ± 13.01	**5.6e-13**	$<$ **1e−16**	**1.3e-06**
	2000 seqs.	RMSD	5.21 ± 1.30	3.93 ± 1.48	2.85 ± 1.08	2.86 ± 1.12	$<$ **1e−16**	$<$ **1e−16**	$<$ **1e−16**
		TM-score	0.34 ± 0.15	0.53 ± 0.22	0.70 ± 0.20	0.69 ± 0.20	$<$ **1e−16**	$<$ **1e−16**	$<$ **1e−16**
		pLDDT	34.59 ± 6.67	48.86 ± 15.22	65.59 ± 12.74	65.41 ± 13.08	$<$ **1e−16**	$<$ **1e−16**	$<$ **1e−16**
	Helix	RMSD	3.23 ± 1.17	3.02 ± 1.12	2.92 ± 1.14	2.81 ± 1.09	$<$ **1e−16**	$<$ **1e−16**	**2.1e-08**
		TM-score	0.60 ± 0.20	0.68 ± 0.20	0.65 ± 0.21	0.71 ± 0.19	$<$ **1e−16**	$<$ **1e−16**	**1.4e-12**
		pLDDT	59.77 ± 14.46	60.31 ± 13.52	66.43 ± 13.05	65.37 ± 12.75	$<$ **1e−16**	$<$ **1e−16**	**1.6e-13**
	Sheet	RMSD	3.04 ± 1.20	3.08 ± 1.12	2.89 ± 1.17	2.82 ± 1.08	**1.0e-16**	$<$ **1e−16**	**4.9e-09**
		TM-score	0.67 ± 0.20	0.66 ± 0.20	0.70 ± 0.19	0.70 ± 0.20	$<$ **1e−16**	$<$ **1e−16**	**2.4e-07**
		pLDDT	60.29 ± 14.11	60.14 ± 13.66	63.77 ± 13.53	66.21 ± 12.57	$<$ **1e−16**	$<$ **1e−16**	$<$ **1e−16**
