# Supplementary material for: Trainable subnetworks reveal insights into structure knowledge organization in protein language models
Source: PLoS Comput Biol. 2026 Feb 9;22(2):e1013925. doi: 10.1371/journal.pcbi.1013925 (PMC12928587; doi:10.1371/journal.pcbi.1013925)
Supplement: Attachment — Submitted filename: plm_subnetworks_revision_rebuttal.pdf [file pcbi.1013925.s016.pdf]

We thank the reviewers for their comments and suggestions. We appreciate the time and effort taken to evaluate our work and are grateful for the suggestions that have helped us to improve our submission.

Overview of changes

We have carefully considered each point raised and have addressed them thoroughly in the revised manuscript. Following the helpful feedback from reviewers, we have now:

(1) Re-ran all ESM-2 experiments with the mask learned over all layers. The results and interpretation are the same as previously masking over layers 6 through 30, i.e., subnetworks are successfully learned at all CATH levels (with greater suppression at class and superfamily levels), and structure prediction accuracy with the ESMFold folding trunk is more significantly decreased in suppressed inputs relative to maintained inputs.

(2) Assessed the stability of the mask learning procedure in our approach by training multiple instances of the same ESM-2 650M subnetworks and confirming that they converge to the same performance.

(3) Applied our subnetwork approach to three additional protein language models with varying sizes and architectures, but each trained on UniRef data ProtBERT-UR100 420M (Elnaggar et al. 2021, *IEEE Trans Pattern Anal Mach Intell*), a transformer masked language model with a BERT architecture; CARP-640M (Yang et al. 2024, *Cell Systems*), a convolutional neural network masked language model where transformer layers are replaced by ByteNet dilated CNN blocks; and Dayhoff-170M-UR90 (Yang et al. 2025, *bioRxiv*), a hybrid state-space-model transformer trained with an autoregressive objective.

(4) Added additional analyses on the interpretation of the learned mask(s).

(5) Revised the Discussion section to include implications of subnetwork existence across diverse state-of-the-art PLMs and relate these findings back to existing literature.

We copied the reviewers' comments in full and listed them in blue. Below, we respond to each comment in detail. Direct quotes from our revision are given in ***bold italics***. We are very grateful for the constructive criticism and do agree that addressing these concerns has improved our manuscript.

Reviewer #1 (Remarks to the Author):

Vinod et al. present a method demonstrating how structural information is factorized within the large, sequence-based protein language model ESM2 using trainable subnetworks. By comparing both perplexity changes within ESM2 and alterations in structure prediction performance with ESMFold, the authors provide clear evidence that ESM2 encodes structural elements into distinct subnetworks. Masking these subnetworks significantly perturbs protein structure predictions, underscoring the validating the approach.

The study provides valuable a framework to interpret pLMs to gain insights into the internal workings and limitations. Overall, the training procedures, datasets, and evaluation methodologies are clearly described and executed. However, the manuscript would benefit from additional clarity and development of certain key points (see below). The authors have commendably shared both their code and the training dataset.

We thank the reviewer for the thoughtful assessment and supportive comments about our initial submission. We address the reviewer's major/minor comments below.

Major Comments:

It is unclear why applying the unmodified ESMFold trunk to altered embedding spaces of the subnetwork remains a valid evaluation approach. Although including subnetworks trained to suppress random inputs partially addresses this concern, a discussion or additional analysis (such as an ablation experiment) would help to justify the choice.

We appreciate the reviewer for bringing up this important point. We treat the ESMFold folding trunk as a frozen model that maps sequence representations to atomic coordinates. The full ESMFold pipeline using the unmodified ESM-2 coupled with the frozen folding trunk therefore serves as our baseline for structure prediction performance. Using the frozen ESMFold trunk allows us to isolate the effect of changes in the sequence representation on structure prediction accuracy. By keeping this "decoder" fixed, we can directly test whether the information encoded in the subnetwork-modified representations remains aligned with what the folding trunk expects for the suppression or maintenance inputs. If subnetwork masking disrupts this alignment, a decrease in TM-score or pLDDT, or an increase in RMSD reflects a loss of structural information within the sequence representation. Consequently, if subnetwork-induced perturbations selectively impair structure prediction for particular CATH categories or residue types, this suggests that those structural features were localized within PLM weights that the subnetwork learned to zero out. This evaluation is conceptually analogous to using a pretrained linear probe to investigate the interpretability of a natural language model: the goal is not to re-optimize downstream performance, but to quantify how much biologically meaningful information remains accessible in the subnetwork sequence representations.

To contextualize these results, we train subnetworks that suppress randomly selected sequences. Suppressing random sequences forces the model to mask weights associated with general features, since these inputs do not share any strong structural features that would group them together in the CATH taxonomy. This effectively scrambles the learned representations and should produce a reduction in structure prediction accuracy across both suppression and maintenance inputs—which is exactly what we observe. In contrast, if structural categories are truly factorized within PLM weights, we would expect selective degradation in structure prediction for the targeted suppression categories but not for the random subnetworks. We observe this as well. Since ESMFold acts as a pretrained decoder from representation space to structure, these selective effects reveal where structural knowledge is encoded in the original PLM weights.

We now write the following to clarify our decision to use ESMFold in the revised manuscript (see new lines 259-266):

ESMFold (Lin at al. 2023, Science) introduced a folding trunk–-a simplified version of AlphaFold2's Evoformer (Jumper et al. 2021, Nature)---that converts language model representations into 3D structures. Using this frozen trunk allows the isolation of subnetwork-induced changes in sequence representations and enables investigation into how they affect structure prediction accuracy. Since the folding trunk serves as a fixed decoder, any degradation in template modeling (TM) score or predicted local distance difference test (pLDDT), or increased root mean square deviation (RMSD), directly reflects a loss of relevant structural information in the sequence representations.

We then detail our experimental results where we train subnetworks that suppress randomly selected sequences in new lines 227-241, with corresponding results being displayed in Fig. S2 and Table S2:

As a control, we trained two types of subnetworks. The first control was to suppress N randomly selected sequences, where values of N were chosen to mimic the size of a CATH sequence category. The second control was to suppress randomly selected residues. We evaluated the residue control subnetwork separately on alpha helices and beta sheets. Both the random sequence and random residue subnetworks fail to produce differential performance on the suppressed inputs, with the only exception being the sequence control with $N = 2000$, which resulted in a 2.6-point increase in perplexity on suppression inputs relative to ESM-2 (8.8). In contrast, subnetworks trained to suppress CATH-Class level categories of sequences are able to attain a perplexity of greater than 30, while achieving maintenance goals. We reasoned that any increase in perplexity in the sequence-controls arises because the mask learning process removes parameters that encode features broadly shared across sequences. Since randomly selected sequences do not share meaningful structural characteristics, the subnetwork cannot identify parameters specific to suppressed structural information and instead must zero out weights important for general MLM performance, which can lead to a uniform increase in perplexity across all inputs.

Surprisingly, suppressing random positions had a substantial negative impact on performance, I would have expected that this control would overall maintain performance. A clear discussion of this unexpected outcome and its implications is needed.

This is an important point, and we apologize for not being clearer in our initial submission. As we mentioned in response to the reviewer's previous comment, in the revised manuscript, we now clarify that our random suppression controls are performed separately at sequence level and at the residue level. The residue control, i.e., suppressing random positions, are now presented via hatched markers In Figures 2C, and 3B-C for clarity. The residue control maintained performance in the MLM evaluation across all types of inputs. For sequence-level suppression, we trained subnetworks to suppress randomly selected sets of sequences (N = 100, 200, 1000, and 2000), with N chosen to mimic the sizes of the CATH categories. In the language modeling results (Fig. 2B-C and Table S4), these random subnetworks do not achieve selective increases in perplexity on the suppressed inputs while still achieving ESM-2 perplexity on the maintenance inputs. The suppression of 2000 random sequences causes a small drop in MLM performance across both suppression and maintenance inputs. This is expected: since the randomly chosen suppressed sequences do not share any structural characteristics (i.e., they do not belong to the same category as defined in the CATH taxonomy), the subnetworks cannot identify common parameters that are responsible for encoding structural information about these sequences and instead zero out PLM weights that are important for general performance.

In the structure prediction analysis (Fig. 3B-C), subnetworks trained to suppress large random sets of sequences cause a large increase in RMSD on both suppression (up to 2.5 Å) and maintenance inputs (greater than 1 Å). In contrast, non-control subnetworks (i.e., those applied to sequences corresponding to the same categories) show large increases in RMSD on the suppression inputs (up to 4 Å), and less than a 0.25 Å increase in RMSD on maintenance inputs (on average). This same trend is seen for TM score and pLDDT (Fig. S4). Indeed, random suppression tends to reduce accuracy broadly, as the reviewer points out. However, this is expected: the ESMFold folding trunk relies on relevant structural information in the sequence representations (which we show to be factorizable via PLM weights in Fig.

2C), and random suppression disrupts that organization. When subnetworks trained on random sequences zero out parameters that contribute to broadly useful features, the resulting representations become scrambled and lose coherence with what the folding trunk expects. As a result, the input embeddings no longer contain sufficient structural information to enable accurate structure predictions, leading to a noticeable decrease in prediction accuracy across both suppression and maintenance inputs.

Again, we detail our experimental results where we train subnetworks that suppress randomly selected sequences in new lines 227-241, with corresponding results being displayed in Fig. S2 and Table S2.

The subnetworks were trained specifically on single-domain proteins annotated with CATH annotations. It would be important to show how subnetworks generalize to multi-domain proteins that combine suppressed and maintained domains.

We appreciate the reviewer for making this great suggestion to examine multi-domain proteins. Our study intentionally focuses on single-domain proteins annotated by CATH because these provide clear structure-label mappings that allow subnetworks to learn and isolate representations specific to well-defined structural categories. Including multi-domain proteins would introduce overlapping or mixed annotations (i.e., sequences containing both suppressed and maintained domains), which would obscure the definitions and interpretability of performance on the suppression and maintenance criteria.

We incorporate this suggestion as a future direction of our work in the Discussion (lines 377-378):

While assessing generalization to multi-domain proteins and complexes is an interesting future direction, the goal of this work is to characterize how structural categories are factorized in PLM weights at the level of their basic, clearly labeled secondary structure components.

Currently it is not possible to install the code. While installing dependencies according to the GitHub instructions, dependency conflicts occurred with Python 3.10 and 3.11 at the step:

error message with python3.10: ERROR: Ignored the following versions that require a different python version: 1.3.0 Requires-Python >=3.11; 1.3.3 Requires-Python >=3.11; 1.4.0 Requires-Python >=3.11; 2.3.0 Requires-Python >=3.11; 2.3.1 Requires-Python >=3.11; 2.3.2 Requires-Python >=3.11; 3.5 Requires-Python >=3.11; 3.5rc0 Requires-Python >=3.11
ERROR: Could not find a version that satisfies the requirement plmprobe==0.1 (from versions: none)
ERROR: No matching distribution found for plmprobe==0.1

error message with python 3.11: ERROR: Ignored the following versions that require a different python version: 1.21.2 Requires-Python >=3.7,<3.11; 1.21.3 Requires-Python >=3.7,<3.11; 1.21.4 Requires-Python >=3.7,<3.11; 1.21.5 Requires-Python >=3.7,<3.11; 1.21.6 Requires-Python >=3.7,<3.11
ERROR: Could not find a version that satisfies the requirement plmprobe==0.1 (from versions: none)
ERROR: No matching distribution found for plmprobe==0.1

All was tested in a conda environment with Python 3.10 as well as 3.11. See the installation instructions:
pip install --extra-index-url https://download.pytorch.org/whl/cu121 -r
environments/h100env_requirements.txt

We thank the reviewer for thoroughly assessing our work, and we apologize for this error that arose from an unrelated dependency. We have updated the code repository to resolve this as well as to include the

code for all new experiments introduced in our revised submission (please see the changes in the GitHub at https://github.com/microsoft/plm_subnetworks).

Minor Comments:

Please briefly explain why only ESM-2 650M was chosen. Showing whether the observed performance degradation generalizes to other pPLMs would strengthen the analysis.

This is a great suggestion. In addition to being one of the most prevalent PLMs, ESM is unique because it has an associated ESMFold structure module, which other widely used PLMs do not have. This enabled us to directly probe structure-related information from language modeling representations. We chose ESM-2 650M because it is widely adopted and well-studied in the academic community, offering strong performance on both language modeling and structure prediction tasks with reasonable computational cost. Learning a mask over model parameters effectively doubles GPU memory usage, and the 650M variant allowed us to train each subnetwork efficiently on a single H100 machine. Smaller models yield less reliable structure prediction performance, while larger models would be prohibitively expensive for the full set of experiments. Because the performance of ESM-2 on both language modeling and structure prediction has been shown to scale with model size (Lin et al. 2023, *Science*), we expected the subnetworks to exhibit similar scaling trends.

To strengthen our conclusions with regard to other PLMs, in the revised manuscript, we now apply our subnetworks approach to three additional models with varying size and architectures: ProtBERT-UR100 with (Elnaggar et al. 2021, *IEEE Trans Pattern Anal Mach Intell*), a 420M parameter transformer masked language model with a BERT architecture; CARP (Yang et al. 2024, *Cell Systems*), a 640M parameter convolutional neural network masked language model where transformer layers are replaced by ByteNet dilated CNN blocks; and Dayhoff-170M-UR90 (Yang et al. 2025, *bioRxiv*), a 170M parameter hybrid state-space-model transformer trained with an autoregressive objective. In our new results, we find that all models show similar behavior to what we saw with ESM-2 and factorize sequence representations more than residue representations. The subnetworks also achieve these results with less than 3% sparsity of model parameters, as seen in ESM-2 (see the new Fig. S3).

We clarify the choice of ESM-2 650M in the revised Discussion (see new lines 337-346):

We chose ESM-2 for our main analyses because in addition to it being widely adopted and well-studied, it has an associated structure module that allows us to directly probe structure information in language modeling representations. We chose the 650M variant because it offers strong performance on both language modeling and structure prediction related tasks with reasonable computational cost. Learning a mask over model parameters effectively doubles GPU memory usage, and the 650M variant allowed us to train each subnetwork efficiently on a single H100 machine. Smaller ESM-2 variants yield less reliable structure prediction performance, while larger variants would be prohibitively expensive for the full set of experiments we presented. Since the performance of ESM-2 has been shown to scale with model size (Lin et al. 2023, Science; Li et al. 2024, ICML 2024), we expect the subnetwork behavior observed here to exhibit similar scaling trends.

We also now detail results from running subnetworks with other PLMs in the revised Results (see new lines 242-252):

We also applied our subnetworks approach to three additional PLMs of varying size, architectures, and pretraining tasks, each having been trained on UniRef data: ProtBERT-UR100 (420M parameters) (Elnaggar et al. 2021, IEEE Trans Pattern Anal Mach Intell), a transformer masked language model with a BERT architecture; CARP-640M (640M parameters) (Yang et al. 2024, Cell Systems), a convolutional neural network masked language model where transformer layers are replaced by ByteNet dilated CNN blocks; and Dayhoff-170M-UR90 (170M parameters) (Yang et al. 2025, bioRxiv), an efficient hybrid state-space-model transformer trained with an autoregressive objective. We present these analyses as additional results in Fig. S3. The subnetworks in these additional PLMs show similar trends to ESM-2, in that they are discovered by pruning less than 3% of model parameters, discover the strongest factorizations at the Class and Homologous Superfamily levels, and factorize categories of sequences to a larger extent when compared to residues.

Currently it is not clear why the MLM maintenance training objective is necessary in addition to the "maintenance goal". As far as I understand, the maintenance goal has the purpose of keeping performance intact on a set of inputs to be compared with those suppressed in the suppression objective, but naming both objectives "maintenance" makes the latter seem redundant. This needs clarification.

We thank the reviewer for drawing our attention to this point of confusion. With respect to the maintenance goal, two things are important. First, the subnetwork predictive distribution over the maintenance inputs mimics that of the pretrained model. Second, the subnetwork is able to maintain MLM performance on the maintenance inputs.

We followed prior work that confirmed that all 3 loss terms are required. Bayazit et al., (*EMNLP* 2024) used the same composite loss of suppression-KL, maintenance-KL and maintenance-MLM for their application of subnetwork discovery. They performed ablations showing that if either maintenance-KL or maintenance-MLM was not included in the final loss, then the perplexity on the maintenance inputs would increase. In our application, the maintenance-KL objective in Eqs. (3) and (4) ensures that the subnetwork reproduces the predictive behavior of the original model on maintenance inputs: it constrains the subnetwork to remain close to the pretrained ESM-2 model's logits (output distributions) for those sequences. This encourages the subnetwork representations on maintenance inputs to mimic those of ESM-2 on the same inputs. However, in addition to mimicking logits of the original PLM, the subnetwork is also trained to assign the correct log probability of the correct token via the maintenance-MLM goal in Eqs. (5) and (6). We now clarify this in the Methods section (see lines 107-117):

In practice, the maintenance-KL goal is insufficient because it only enforces similarity between the subnetwork and the original PLM output distributions, without ensuring that the subnetwork retains the ability to assign the correct probabilities to predicted tokens. Prior work demonstrated via a set of ablations that all 3 loss components are necessary to achieve the desired subnetwork behavior on suppression and maintenance inputs Bayazit et al., (EMNLP 2024), and found that omitting either maintenance-KL or maintenance-MLM increased perplexity on the maintenance inputs. We therefore introduce an additional maintenance-MLM loss, which ensures that the subnetwork can still allocate the appropriate probability mass to the right corresponding tokens, preserving its overall language modeling behavior.

Figure 2A under the "Perplexity" subtitle there is a tiny red cross inside a square, seems left there by mistake.

We apologize for this error, and it has been removed in the revised manuscript.

Even though the dataset is not that large, distributing sequences in .fasta format and structures in .pdb format seems a bit inefficient. Please consider providing a compressed version.

The data is uploaded on Hugging Face https://huggingface.co/riavinod/plm_subnetworks which efficiently manages storing model weights and data.

Reviewer #2 (Remarks to the Author):

This study investigates how protein language models (PLMs) internally organise and “factorise” knowledge of protein structural features. It focuses on ESM-2 and introduces trainable subnetworks as a method to probe the model’s learned parameters for structure-specific information. In practice, the authors define suppression sets of sequences belonging to a specific structural class using CATH hierarchical categories and secondary structure labels and maintenance sets for all other sequences. By applying a multi-objective training, they obtain sparse subnetworks of the original model specialised to each structural concept. A total of 36 such subnetworks were trained, covering multiple levels of structure granularity. The authors then assess how removing or isolating these structural knowledge subnetworks affects downstream 3D structure prediction using ESMFold.

The study finds that certain structural features in the PLM are indeed encoded in a factorised manner. The model can predominantly disentangle extremely coarse-grained structural or very fine-grained ones, whereas intermediate-level categories are not cleanly separable. The authors present this subnetwork approach as a new framework to study feature entanglement in biological language models and suggest it could be used to guide models toward more interpretable representations. Overall, this is an original study that provides valuable insights into the modularity of learned representations in pLMs. The paper is a valuable contribution to both the machine learning and protein science communities. However, there are also important limitations and open questions, which should be addressed.

We thank the reviewer for the kind words, and we appreciate them for taking the time to evaluate our work.

Major Comments:

A key limitation is that the analysis is performed on only one PLM (ESM-2). While ESM-2 is a high-quality model, the conclusions drawn - that structural knowledge is factorised in certain ways - may not universally hold for other architectures or training methods. For example, a different protein language model (such as ProtT5) might organise information differently. The study would be stronger if the authors at least discussed this limitation thoroughly or, ideally, provided some evidence on an additional model to show whether the phenomenon is consistent. As it stands, readers would automatically assume that the findings reflect properties of all large PLMs, which is an extrapolation not directly tested. This is a significant issue because the claim about how PLMs factorize structural categories could be model-specific.

We appreciate the reviewer’s suggestion to broaden the scope of the pretrained PLMs we consider in our work. In the revised manuscript, we now apply our subnetworks approach to three additional state-of-the-art models with varying size and architectures: ProtBERT-UR100 (Elnaggar et al. 2021, IEEE

Trans Pattern Anal Mach Intell), a transformer masked language model with a BERT architecture; CARP-640M (Yang et al. 2024, Cell Systems), a convolutional neural network masked language model where transformer layers are replaced by ByteNet dilated CNN blocks; and Dayhoff-170M-UR90 (Yang et al. 2025, bioRxiv), an efficient hybrid state-space-model transformer trained with an autoregressive objective. In our new results, we find that all models show similar behavior to what we saw with ESM-2 and factorize sequence representations more than residue representations. The subnetworks also achieve these results with less than 3% sparsity of model parameters, as seen in ESM-2 (see the new Fig. S3). Note that the downstream structure prediction analysis was not repeated here due to the lack of a pretrained folding trunk compatible with these models. Overall, we believe that these analyses further strengthen our conclusions about subnetwork behavior existing across PLMs trained on UniRef data as they cover a range of model sizes, architectures, and pretraining tasks.

We also now detail results from running subnetworks with other PLMs in the revised Results (see new lines 242-252):

We also applied our subnetworks approach to three additional PLMs of varying size, architectures, and pretraining tasks, each having been trained on UniRef data: ProtBERT-UR100 (420M parameters) (Elnaggar et al. 2021, IEEE Trans Pattern Anal Mach Intell), a transformer masked language model with a BERT architecture; CARP-640M (640M parameters) (Yang et al. 2024, Cell Systems), a convolutional neural network masked language model where transformer layers are replaced by ByteNet dilated CNN blocks; and Dayhoff-170M-UR90 (170M parameters) (Yang et al. 2025, bioRxiv), an efficient hybrid state-space-model transformer trained with an autoregressive objective. We present these analyses as additional results in Fig. S3. The subnetworks in these additional PLMs show similar trends to ESM-2, in that they are discovered by pruning less than 3% of model parameters, discover the strongest factorizations at the Class and Homologous Superfamily levels, and factorize categories of sequences to a larger extent when compared to residues.

The finding that intermediate structural categories cannot be cleanly disentangled by any subnetwork is intriguing, but the paper does not thoroughly probe why this is the case. This is a major interpretative gap. The authors report the outcome that subnetworks for medium-grained classes don't achieve the same selective suppression, but they stop short of analysing the causes or implications. Is it because the model inherently entangles those features (perhaps due to overlapping sequence patterns or evolutionary signals)? Or could it be due to the definition of those categories being broad or heterogeneous? Currently, one might feel the authors identified a limitation (PLM knowledge not factorised at mid-level) but did not leverage it to provide deeper insight. Without such exploration, the treatment of this result is a bit superficial. This is an important issue because it touches on the limits of the model, acknowledging and examining it in detail would enhance the scientific soundness of the work.

We thank the reviewer for highlighting this important point. The reduced separability of intermediate CATH categories likely reflects both biological and representation factors rather than a limitation of the subnetwork approach, as all subnetworks exhibit this behavior of maximizing suppression on the class and superfamily classifications. We detail some of these factors below.

First, the CATH hierarchy itself is non-uniform in granularity: (i) “Class” categories are defined by coarse secondary structure content (e.g., mainly Alpha, mainly Beta, Alpha-Beta); (ii) the “Homologous Superfamily” captures fine-grained evolutionary relationships; and (iii) intermediate levels such as “Architecture” and “Topology” aggregate proteins that share only partial structural motifs. Many of these categories are structurally heterogeneous, containing folds with distinct secondary structure

arrangements but similar overall geometry. This heterogeneity makes them more difficult to represent as separable subspaces within a single PLM.

Second, the structure prediction statistics of these categories support this interpretation. In our structure prediction analysis (Fig. 3C), mixed Alpha-Beta proteins show the largest degradation in RMSD when using subnetwork representations, followed by Beta and then Alpha class proteins. This mirrors the inverse trend in ProteinMPNN perplexities (Table S5), where Alpha-Beta proteins have the lowest perplexity (i.e., the most sequence-constrained and least tolerant to residue substitutions) and alpha proteins have the highest. These results imply that the more structurally constrained and compositionally mixed folds occupy more tightly organized, overlapping regions of PLM embedding space. Small perturbations to these shared representations disrupt ESMFold's performance most strongly, highlighting that entanglement arises from the inherent coupling between Alpha-Beta (mixed) motifs across multiple fold families, not from instability in the subnetwork training.

Together, these findings indicate that the degree of factorization mirrors the natural compositional hierarchy of protein structure: global features (secondary structure content) and local evolutionary features (Homologous Superfamily) are cleanly separable, while intermediate features (defined by more abstract geometric relationships) are distributed across overlapping representational subspaces. This pattern suggests that PLMs encode protein structure in a partially entangled but biologically meaningful way, consistent with the compositional and continuous nature of protein folds. Importantly, this behavior is observed across all models we tested, each of which cleanly separates coarse (Class) and fine-grained (Homologous Superfamily) categories, and most successfully factorizes Alpha-Beta (mixed) proteins.

In the revised manuscript, we now provide this explanation in the Results (see new lines 212-220):

One possible explanation for suppression being most effective at the Class and Homologous Superfamily levels is that these categories represent the most distinct structural and evolutionary boundaries in protein sequence space. Coarse Class categories capture global, fundamental secondary structure composition (i.e., mainly Alpha, mainly Beta, or ``mixed'' Alpha-Beta classes), while fine-grained superfamilies reflect localized evolutionary relationships, both of which have been shown to form well-separated regions in PLM embedding space (Rives et al. 2021, PNAS; Alley et al. 2019, Nature Methods; Belper and Berger 2019, CoRR). In contrast, intermediate categories such as Architecture and Topology are structurally heterogeneous, combining folds that share only partial motifs but differ in geometry, making them inherently less separable and thus more difficult for subnetworks to isolate into distinct groups.

Although the training objective explicitly tries to preserve performance on maintenance inputs, the results show that even those inputs' structure predictions were statistically significantly perturbed by the subnetwork masking. This implies that the subnetworks, while largely maintaining perplexity on non-suppressed sequences, still altered the representations in a way that affects downstream tasks broadly. From a methodological standpoint, this is a concern: the intention was to localise and remove only the knowledge relevant to one class, yet it proved impossible to do so without collateral damage to other classes' predictions. In other words, the PLM's knowledge of one structural category is not perfectly modular - some of it is shared or intertwined with other categories. The authors do note this outcome, but a criticism is that the paper doesn't propose or investigate solutions or deeper implications of this entanglement. For example, could adjusting the loss weighting (making the maintenance goal stronger) produce a "cleaner" separation at the expense of less suppression? This issue is significant because it shows a limitation in the subnetwork approach's efficacy: the subnetworks cannot isolate one concept

without affecting others, which slightly undermines the claim that structural categories were found to be factorised. It would be valuable for the authors to discuss this point in depth.

We thank the reviewer for pointing out this important result for discussion. First, we want to reemphasize that our goal in this work is to uncover factorizations within the weight space of protein language models (PLMs). We consider this successful based on the perplexity results, which demonstrate that subnetworks can selectively degrade language modeling performance on suppression inputs while maintaining ESM-2 perplexity on the corresponding maintenance inputs. The training objective is explicitly designed to preserve representational fidelity in sequence space, and we therefore evaluate subnetwork performance based on perplexity, following standard practices for benchmarking PLM quality.

To evaluate how this representational factorization propagates to downstream tasks, we use the frozen ESMFold folding trunk as a structural readout of PLM representations. The ESMFold folding trunk can be viewed as a separate pretrained model that maps sequence embeddings to atomic coordinates. Because it is fixed and not jointly optimized with the subnetwork, any localized suppression in the PLM weight space can propagate into measurable differences in predicted structures. We find that the maximum change in RMSD on maintenance inputs is 0.31 Å, which is extremely small and only observed for the largest suppressible category, CATH Class "Alpha-Beta" (N = 3129), while suppression inputs show RMSD increases of up to 4 Å. Similar trends are observed for TM-score and pLDDT (Fig. S4). These results indicate that subnetwork masking produces targeted and category-specific perturbations in the representations, rather than globally disrupting structure prediction performance.

Importantly, the small but statistically significant changes in maintenance-input structure predictions indicate that structural information in ESM-2 is not perfectly modular only when projected out to spatial coordinates via the ESMFold folding trunk. The folding trunk remains sensitive to representational shifts that indirectly affect suppressed categories, reflecting the distributed nature of structural knowledge across PLM weights. Supporting this, the magnitude of structural perturbation follows a systematic trend: mixed alpha-beta proteins are most affected, followed by beta, and then alpha classes. ProteinMPNN perplexities computed on these same CATH categories (Table S5) show that mainly alpha proteins have higher perplexities (implying greater residue-level permissibility) while alpha-beta proteins have the lowest, indicating more constrained sequence-structure mappings. Consequently, alpha-beta proteins, which occupy more specific and compositionally mixed regions of representation space, are more sensitive to embedding perturbations. This relationship explains why small representational shifts cause measurable, though minor, changes in ESMFold predictions even on maintenance inputs: the perturbation reflects true biological and representational coupling between categories that are only uncovered by ESMFold and structure prediction, and not by ESM-2 sequence perplexities. We thus do not view this as a failure of factorization in PLM weights, but rather this occurs because ESMFold integrates multiple structural signals when mapping embeddings to coordinates.

More broadly, these findings suggest that masked language modeling performance alone is insufficient to fully assess representation quality. While perplexity captures how well a model predicts sequence-level statistics, downstream structural evaluations reveal how subtle representational changes propagate through learned, distributed encodings of biological information.

We discuss this in the newly revised Results section (lines 282-291):

We reason that the combination of pronounced effects on suppression inputs and minimal changes in structure prediction accuracy on maintenance inputs---together with the significant p-values---indicates that the frozen ESMFold trunk is sensitive to subtle representational shifts,

rather than reflecting a lack of modularization in sequence representations. The subnetwork mask learning procedure was not optimized to preserve structure prediction accuracy; instead, it was designed to partition the PLM's weight space based on sequence-level characterization and MLM performance. Consequently, small variations in downstream structural metrics are expected, and this analysis mainly serves to reveal how sequence-level factorizations influence structure prediction capabilities.

The approach used (differentiable masking with a multi-term loss) is technically complex, and the paper provides many details (some delegated to appendices) about hyperparameter choices (mask initialisation, mask update schedule, loss weight λ coefficients, etc.). A potential issue here is the sensitivity and stability of this training procedure. The authors do not thoroughly report on how robust the mask learning is - for instance, if one trains the same subnetwork multiple times from different random initial mask values, do we obtain the same set of important weights, or does it vary? There is a concern that the optimisation problem for finding subnetworks is non-convex and could have multiple solutions (or degenerate solutions where the mask fails to converge to a clear separation). If the success of isolating a given structural class depends heavily on hyperparameter tuning or luck in initialisation, that would weaken the reliability of the conclusions. This is a major technical point because it speaks to the rigour of the method: without demonstrating stability, it's hard to know how definitive the identified knowledge-critical weights are.

We thank the reviewer for raising this important point regarding the stability of mask optimization. We want to point out that we initialize each mask parameter logit l_i such that its sigmoid activation corresponds to an initial retention probability $p_{keep,0} = 0.99$. Mathematically, this is expressed as the log odds $l_i = \log(p_{keep,0}/(1 - p_{keep,0}))$, which initializes soft mask scores with nearly all parameters retained. This initialization ensures that the subnetwork begins training from the full model — meaning that the MLM performance on the suppression and maintenance inputs matches ESM-2 baselines for these respective categories of inputs. The mask learning procedure then proceeds to prune parameters over training; therefore, any changes in performance arise from learned suppression and not from a random initialization. Empirically, repeating training with different random seeds yields consistent sparsity patterns and suppression behavior, indicating that the optimization converges to stable, reproducible subnetworks rather than degenerate minima. We report the results of training three separate instances of each CATH Class-level subnetwork in Fig. S2 and Table S2.

With respect to hyperparameters, we standardized the masking learning procedure for each model such that all subnetworks reported in our revised manuscript were trained with the same hyperparameters. When training our additional PLMs (Fig. S3), we conducted new grid searches to identify the optimal hyperparameter combination for each additional PLM. Here, suppression and maintenance-KL and maintenance-MLM lambdas, learning rate, and warm up epochs were fixed for all subnetworks. These hyperparameter configurations are reported in Table S1. Similar to the ESM-2 subnetworks in the main text, we held hyperparameters constant across the subnetworks of each additional model. We include the results of these grid searches for all models in CSVs in the accompanying code and data, which we hope will be useful to those looking to train subnetworks with our framework in the future.

We reference this in the new S1 Text (see Section S1.1):

We set the below hyperparameters to the same values across all subnetworks learned within the same model. We include the results of the grid search on subnetwork performance in the

accompanying code and data (see https://github.com/microsoft/plm_subnetworks for more information). The hyperparameters that vary by PLM are listed below and summarized in Table S1.

The authors choose to apply the trainable mask only to the later layers of the transformer (layers 6-33) based on a prior finding that most structural signals reside in higher layers. While this is a reasonable heuristic to improve training stability, it may introduce a subtle bias. By not allowing any changes to the first 5 layers, the method presupposes that no crucial structural information is encoded there. If that assumption were false, the subnetwork might fail to remove certain knowledge simply because it wasn't permitted to. Early transformer layers typically capture generic features, but the authors should clarify this choice. Ideally, one could test if allowing the mask over all layers changes the results. In other words, the paper aims to find all weights responsible for a given structural category, yet in practice, it only searches in a subset of the network. If important features were in layers 1-5, they would be missed.

We thank the reviewer for raising this point and for the excellent suggestion. In the initial experiments, we applied the trainable mask only to transformer layers 6-33 of ESM-2 (i.e., the top 80% of layers) based on prior analyses showing that higher layers capture most structural information and are learned in superposition (Simon and Zou 2025, *Nature Methods*; Adams et al. 2025, *bioRxiv*). Upon the reviewer's suggestion, we reconsidered our approach in the revised manuscript. Here, we reasoned that if the mask learning were successful, it would automatically learn the relevant layers to mask to achieve selective suppression. We thus trained subnetworks over all 33 layers of ESM-2, and reported the results in the main text (see the new Fig. 2). The results were consistent with those reported previously: suppression and maintenance perplexities, as well as structure-prediction performance, showed similar trends, with the main difference being that subnetworks identified in layers 6-33 were achieved by pruning up to 5% of parameters, but masking all layers only requires up to 3% of parameters to be pruned. We reason that this is because the subnetwork now has a larger representational capacity due to the mask having more parameters. This revision allowed us to infer that early ESM-2 layers contribute minimally to the subnetwork's structural factorization signal. Conceptually, allowing the mask to be implemented over all layers, and seeing similar results as when only masking layers 6-33, confirms that structural knowledge in ESM-2 is concentrated in mid-to-late layers and that the identified subnetworks reflect genuine modular organization rather than artifacts of layer selection. We also plot the mask activation (i.e., layers where most PLM parameters are pruned) and confirm this result (Figs. S5-S8). Importantly, these new results also suggest that the distributed encoding of structure emerges hierarchically, with early layers retaining general sequence priors and later layers integrating those priors into fold-specific representations. While our initial choice to mask layers 6-33 has proved to be valid, allowing the mask over all layers allows us to be more rigorous and removes any potential bias with respect to new models trained when there isn't enough established prior work to suggest which layers might be important. We thus similarly mask over all layers of the additional PLMs we include in the supporting information (Fig. S3).

The study successfully identifies masks that isolate structural categories, but it provides limited interpretation of which parts of the model those masks correspond to. For instance, readers might be curious if certain transformer layers or attention heads are consistently pruned for particular structural classes. As far as I understand, the article does not report any analysis of the mask patterns. Including even a qualitative summary of mask characteristics would strengthen the connection between the subnetwork and the notion of knowledge organisation. Without it, the subnetworks are abstract. We know they exist and affect performance, but not how or where in the model the structural knowledge was stored. As a suggestion, the authors could visualise or summarise the fraction of weights pruned per layer. Currently, the paper's focus is on outcomes of mask application; adding interpretability of the mask itself would enrich the contribution.

We appreciate the reviewer for making another great suggestion. In the Supporting Information of the revised manuscript, we now plot the mean and standard deviation of the percent of model parameters pruned at each layer for ESM-2 650M (Fig. S5), ProtBERT-UR100 (Fig. S6), CARP-640M (Fig. S7), and Dayhoff-170M-UR90 (Fig. S8). Across all PLMs, we find that most parameters are pruned at the CATH Class level (the coarsest classification), and the least parameters are pruned at the Homologous Superfamily level (the finest classification). With respect to ESM-2, there is a noticeable pruning of parameters in layers 12-33 to achieve suppression, suggesting that these layers are most important for encoding structural information, which is aligned with the existing literature (see the discussion in our response to the reviewer's previous comment). Across all subnetworks, the Homologous Superfamily has the lowest standard deviation, suggesting higher confidence of the subnetwork being able to identify and zero out parameters pertaining to small structural categories. The higher standard deviation on the Architecture- and Topology-level suggested that the subnetworks struggle due to variation in size and diversity of folds within the category. The standard deviation is highest in the random subnetworks, suggesting overall uncertainty about parameter masking, while real subnetworks have lower standard deviation.

The manuscript cites many relevant studies in the introduction, which is commendable, but in the discussion of results, it could do more to relate its findings back to those studies. It doesn't affect the validity of the work, but a richer comparison with literature would highlight the study's novelty and also reassure readers that important previous findings have been considered in interpreting the results.

We thank the reviewer for suggesting how we can improve the contextualization of our results. We believe that our work has many connections to existing work, namely:

- Structure prediction accuracy is known to scale with model size. Our findings—that structural categories of sequences are stored within specific subsets of weights—suggest that increasing the number of parameters allows the model to encode a greater diversity of structural properties via subnetworks, thereby improving structure prediction performance as model size increases. If structural features are indeed organized within weight space, larger models naturally offer greater capacity to represent diverse structural categories, providing a mechanistic explanation for the observed decrease in perplexity and corresponding performance gains in ESM-2 and ESMFold with scale (Lin et al. 2023, *Science*).

- Protein language models implicitly capture the statistics of co-evolving residues; thus, the more sequences in their training data, the richer the co-evolutionary correlations they can encode. This property may also explain why sequence-level factorization emerges more clearly than residue-level factorization: the model organizes information according to patterns of covariation across entire sequences rather than isolated positions (Zhang et al. 2024, *PNAS*).

- Consistent with recent mechanistic analyses of superposition in large PLMs, we find that subnetworks predominantly prune parameters in the mid-to-late layers (layers 12-33), suggesting that these layers are responsible for storing higher-order structural and functional abstractions (Simon and Zou 2024, *Nature Methods*; Adams et al. 2024, *bioRxiv*). The statistically significant, yet modest, perturbations observed in structure prediction further indicate that perplexity alone may be an incomplete measure of PLM quality. Although subnetwork factorizations are separable in weight space and maintain ESM-2 perplexity, small differences in embedding organization can propagate into measurable effects on structure prediction, highlighting aspects of representational quality not fully captured by language modeling metrics.

- More broadly, our findings reinforce that protein language models exhibit behaviors analogous to those observed in natural language models, encoding a hierarchical organization of sequence features (Bayazit et al. 2023, *EMNLP*; Liu et al. 2019, *CoRR*; Tenney et al. 2019, *ACL*).

- We emphasize it is essential to understand when these behaviors are beneficial or limiting for biological applications. Evaluating PLM performance solely through perplexity may therefore overlook important dimensions of representational fidelity that govern downstream generalization and structural accuracy.

We have fully revised the discussion section to incorporate these points (see lines 331-393).
